# Linking Enol–Keto Tautomerization Ratios in β‑Diketones to Their Physical Properties Using Computational Quantum Chemistry

**DOI:** 10.1021/acsomega.6c03902

**Published:** 2026-08-13

**Authors:** Julia Gabriel, Jesse Na, Brian Guillermo, Lark J. Perez, Erik P. Hoy

**Affiliations:** Rowan University, 201 Mullica Hill Road, Glassboro, New Jersey 08028, United States

## Abstract

Tautomeric equilibria play an essential role in numerous chemical and biological reactions. Despite having structures that only differ by the position of one hydrogen atom, the chemical properties of prototropic tautomers can vary considerably, and consistently predicting their tautomer ratios remains an unsolved challenge. This project aims to determine the chemical and electronic structure factors that control the equilibrium ratio between the keto and enol forms of prototropic tautomers. We utilize computational quantum chemistry software to analyze the impact of substituent effects, intermolecular interactions, and solvent interactions (implicit and explicit) on the tautomerization ratio.

## Graphical abstract

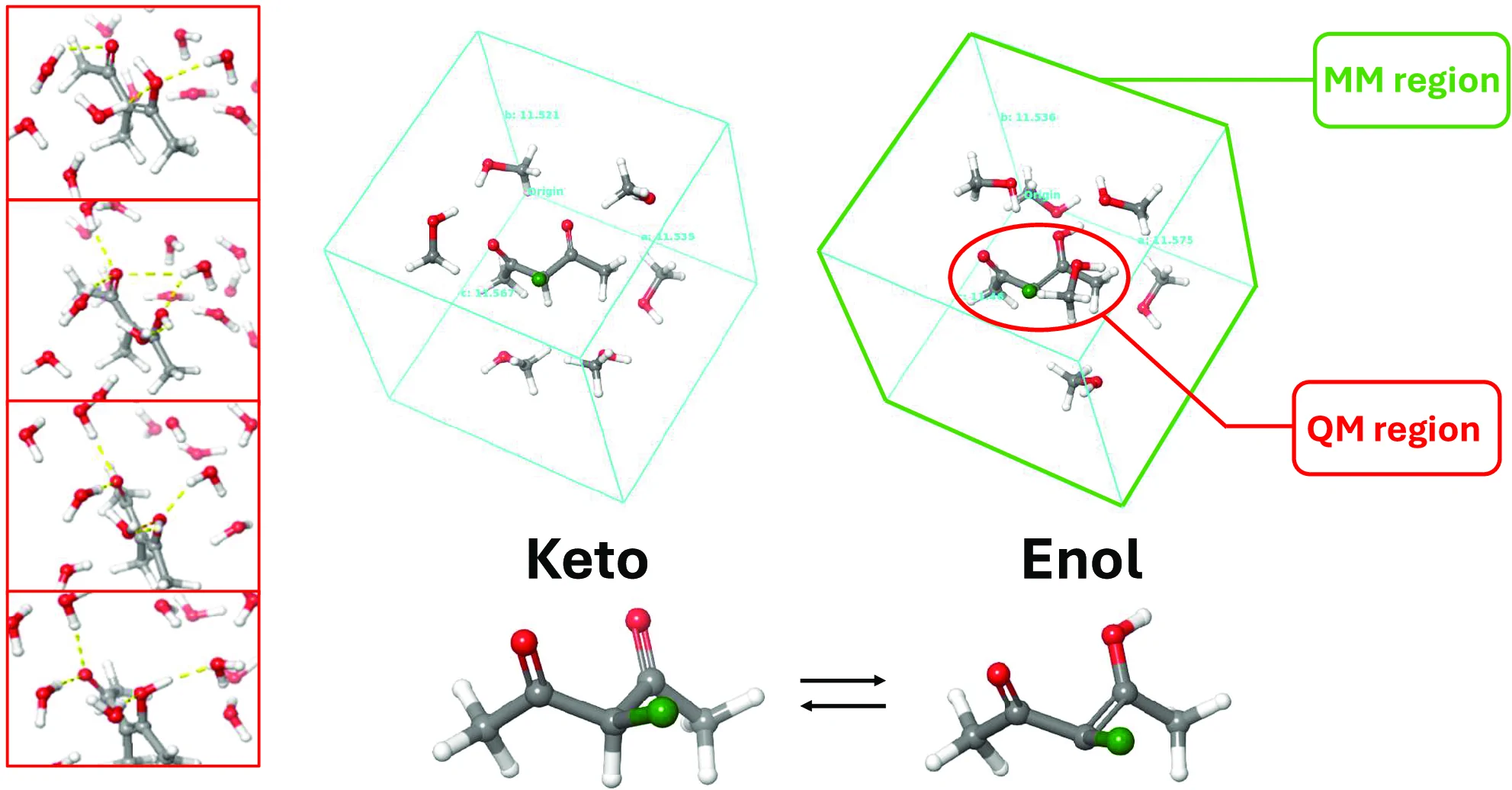

## 1 Introduction

Due to the presence of specific functional groups in a compound enabling tautomeric isomerism, different tautomeric forms of a molecule can display distinct chemical and physical properties significantly impacting biological activity including drug potency, disposition, toxicity, as well as absorption, distribution and drug delivery.^1,2^ Prototropic tautomers in particular are defined as a type of tautomerization in which a proton is transferred from one polar atom to another. In this case, one atom changes from being a putative hydrogen-bond donor to an acceptor while another atom of the same molecule changes from an acceptor to a donor.^3,4^ Accordingly, tautomers in this class differ by the positions of labile proton(s) and π-electrons, and the total number of possible tautomeric forms is an internal property of the compound.^5^ The study of this type of tautomerism is particularly challenging due to the rapid nature of interconversions. In addition, it is well-known that the effects of solvents play a significant role in the tautomeric equilibria since tautomers tend to interconvert between each other via proton interchange mechanisms.^6^ Minor changes to the compound or the environment can significantly alter the ratio of tautomers. These variations complicate the measurement of a compound’s physical properties and the identification of specific bioactive species within a tautomeric mixture.^3^ In the face of these challenges, the lack of a reliable computational protocol for estimating prototropic tautomeric ratios under a range of conditions leads to a correspondingly limited ability to predict or correlate physical and molecular properties with experimental tautomeric ratios in physiologically relevant media.^7–10^ Early semiempirical quantum approaches to tautomerism relied largely on Hückel-type π-electron models, which treated only the π-electron system and were therefore only applicable to aromatic and conjugated tautomers. Rather than determining the quantitative prototropic ratios, these models provided qualitative trends in relative stability due to the neglect of σ-electrons, electron correlation, and explicit proton energetics. Eventually, such π-electron models laid the conceptual groundwork for later, more general semiempirical SCF methods (e.g., CNDO, INDO, MNDO).^11–14^ In recent years, many have sought out other computational means to bypass this issue by creating deep learning models such as DeePMD-kit, TorchANI, and MolTaut.^15–17^ Machine learning methods such as these offer computationally efficient approaches for predicting molecular and physical properties; however, they exhibit notable limitations when applied to tautomeric equilibria such as high sensitivity to small energetic errors, poor attainability of solvent and pH effects, and uncertainty quantification which limits their reliability.^18–20^

As a first step to achieving the goal of developing a reliable computational protocol for estimating tautomeric ratios, we focus on one specific type of prototropic conversion: keto–enol tautomerism. This process occurs in compounds containing a carbonyl (C=O) group and at least one hydrogen atom on an adjacent α-carbon (sp^3^-hybridized), or on another sp^3^-hybridized carbon separated from the carbonyl by a conjugated spacer. During tautomerization, the hydrogen is transferred between the sp^3^ carbon and the carbonyl oxygen, establishing an equilibrium between two forms: the keto and enol tautomers.^5^ In this study, we focused on characterizing this process in 1,3-dicarbonyl compounds, specifically β-diketones. For several decades, tautomerism in β-diketones has received a considerable amount of attention in the realms of inorganic, organic, and physical chemistry. This class of molecules has diverse uses and broad value to science such as organic reagents, catalysts, and chelating ligands with applications in many chemical processes.^21,22^ Within the pharmaceutical, perfume, and cosmetic industries, β-diketones are also used in products such as UV sunscreen and cosmetics are found to exhibit biological activity including antioxidant, anticancer, and antibacterial properties.^23,24^ β-diketones have also been extensively studied in the solid state, both in tautomeric β-diketo or β-keto–enol forms.^25,26^ However, in this setting, the number of β-keto–enol structures surpass the number of β-diketo ones which is attributed to the stabilization of a strong intramolecular hydrogen bond between a proton from a hydroxyl group and an oxygen atom, as well as the presence of two conjugated double bonds.^22^ The steric and electronic properties of the substituents influence the strength of the hydrogen bond, but the main driving forces that shift from β-keto–enol tautomer to β-diketo consist of the electron-withdrawing effect and the steric hindrance of the bulky substituents.^22^

Computationally, the keto form is generally predicted to be more stable than the enol form in neutral systems – primarily due to favorable intermolecular interactions with polar environments that can outweigh the intramolecular effects – the enol tautomer can be favored when additional intramolecular stabilization, such as hydrogen bonding or electron delocalization, is considered.^27–30^ The additional intramolecular interactions decrease the energy of the enol tautomer considerably in comparison to the keto counterpart, favoring the enol form in the gas phase and nonpolar environments.^4,31–33^ The key parameters that can be potentially effect the keto–enol equilibrium include the nature of the substituent and the presence of different solvent models. By further enhancing the understanding behind the question shown above in Figure 1, we examined three compounds (Figure 2) using quantum mechanical methods to determine what computational factors (ex. DFT methods, solvent, and substituent) are correlated with experimental tautomeric ratio predictive accuracy.

**Figure 1:**
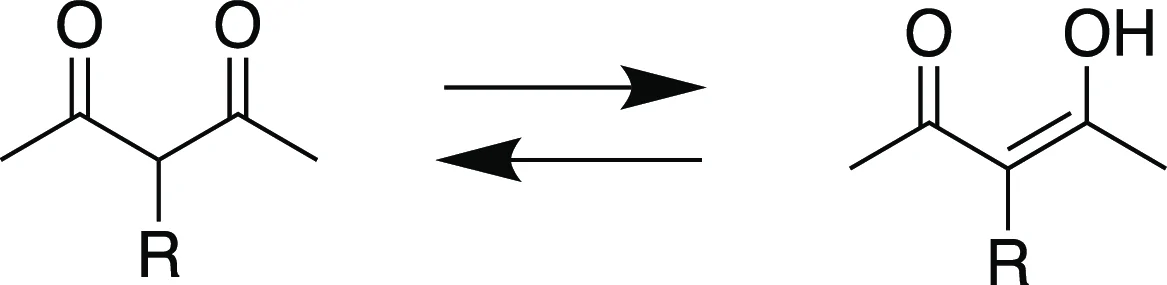
Equilibrium between the keto and enol forms of a molecule is impacted by intrinsic and extrinsic factors.

**Figure 2:**
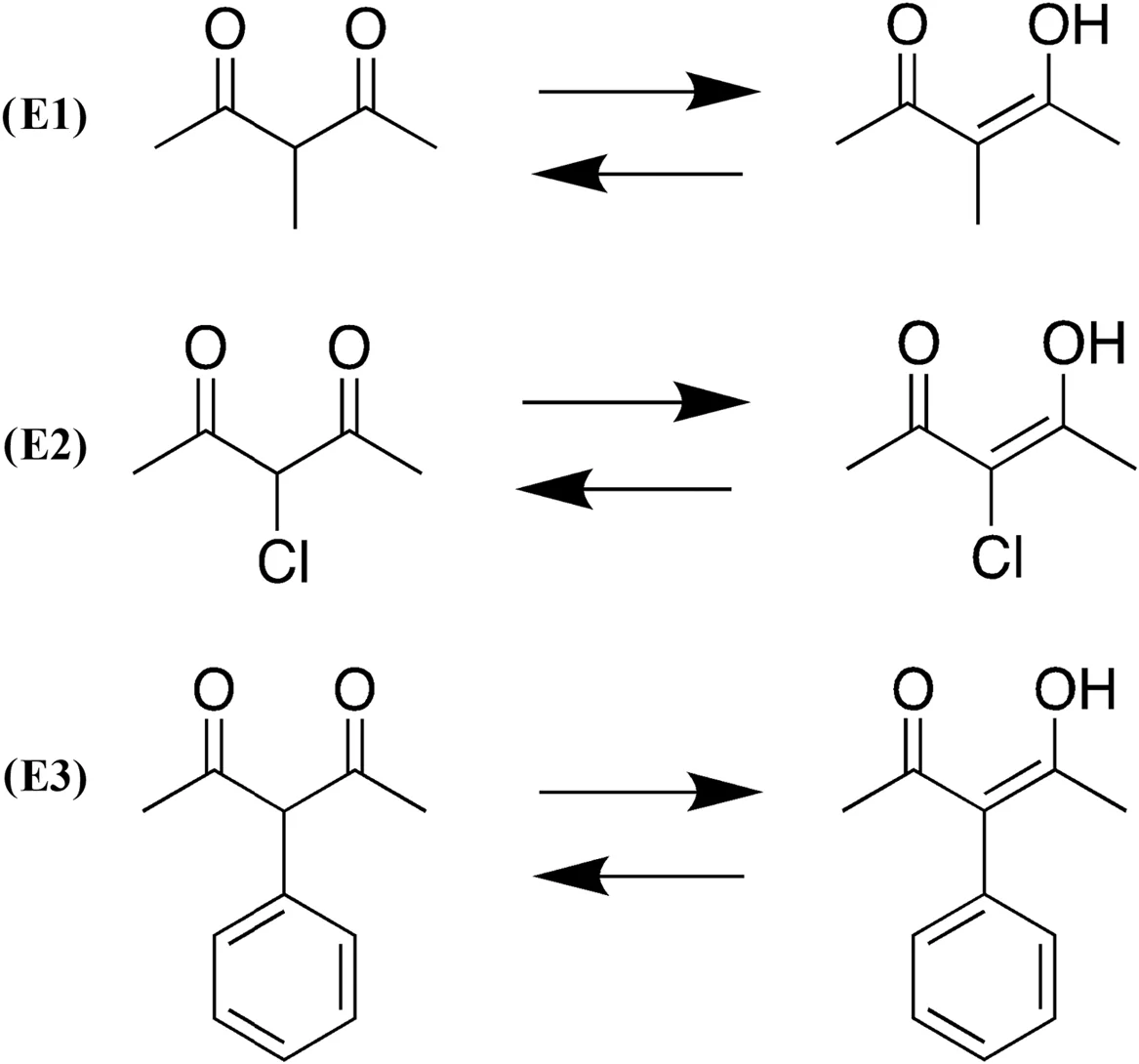
Compounds E1–E3 are used for experimental and computational studies.

We then examined four additional compounds computationally as a second series to demonstrate and characterize some structural features impacting tautomerization to further enhance our understanding of this process (Figure 3).

**Figure 3:**
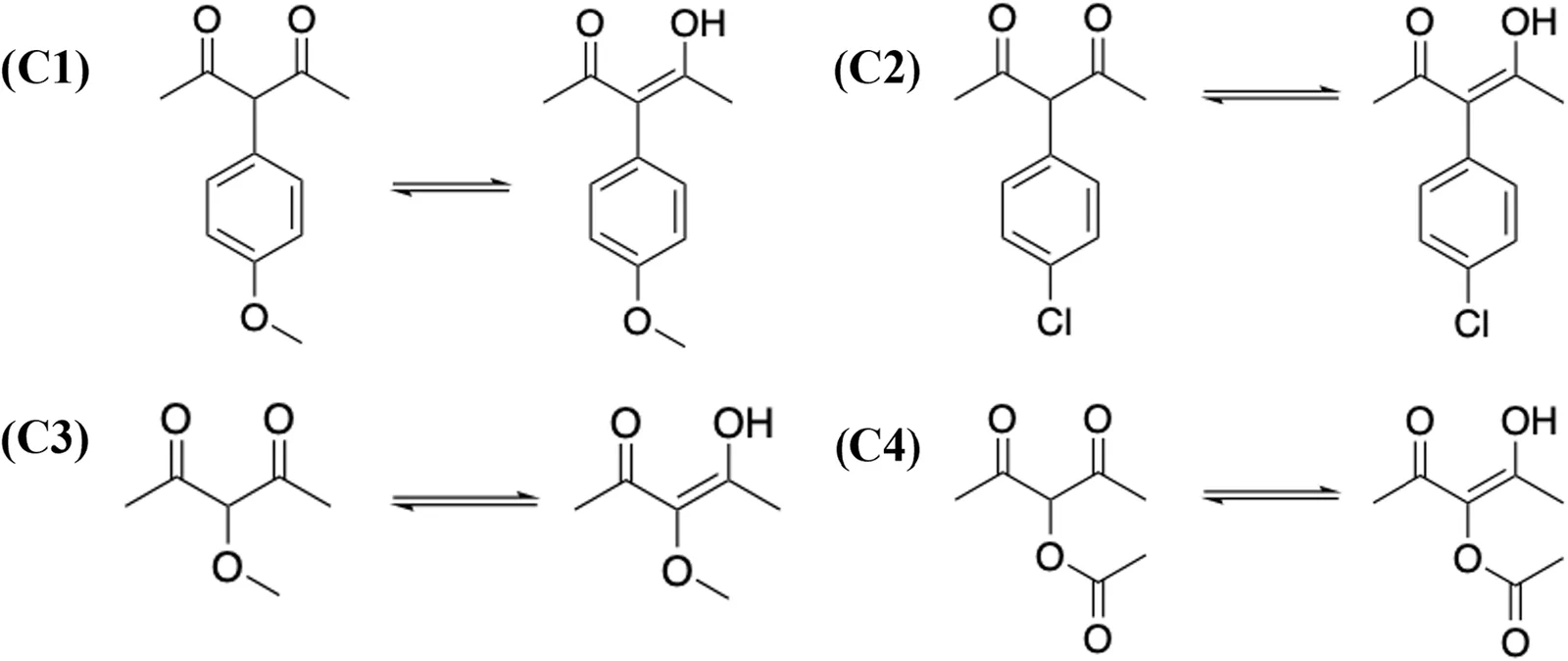
Compounds C1–C4 are used for computational studies.

## 2 Results and Discussion

### 2.1 Experimental Synthesis and Analysis

The keto–enol tautomer ratios for compounds E1–E3 (Figure 2) were determined in the indicated deuterated solvent by integration of characteristic ^1^H NMR signals (see Supporting Information, SI) at ambient temperature. For each compound, ^1^H NMR spectra were collected, and the integration ratio of diagnostic signals was used to determine tautomeric ratio. For 3-methyl-2,4-pentanedione, the α-methyl substituent appears as a doublet (3H, δ 1.1–1.4, depending on solvent) in the keto form while shifting to downfield and appearing as a singlet in the enol form. Integration of these signals was used to provide the tautomeric ration. For 3-chloroacetylacetone, the methylene proton in the keto form (1H, δ 4.6–5.6, depending on solvent) and/or the exchangeable enol proton (1H, δ 15.2–15.6, depending on solvent) enabled identification of the methyl groups corresponding to the enol and/or keto tautomers for this compound. Integration of the methyl singlets (6H, δ 2.1–2.5, depending on solvent) enabled determination of the tautomeric ratio. Finally, for 3-phenyl-2,4-pentanedione, the methylene proton in the keto form (1H, δ 5.3–5.6, depending on solvent) enabled identification of the methyl groups corresponding to the enol and/or keto tautomers for this compound. Integration of the methyl singlets (6H, δ 1.7–2.2, depending on solvent) enabled determination of the tautomeric ratio.

### 2.2 Experimental Results Compared to Implicit Solvent DFT Methods

The combined experimental tautomeric ratios for compounds E1–E3 as determined by ^1^H NMR are presented in Table 1. Compounds E2 and E3 exhibited a high percentage of the enol form while compound E1 displayed an equally distributed percentage between the keto and enol tautomers. Compound E3 is expected to favor the enol form more so than the rest of the compounds due to the conjugation of the phenyl ring.^34–36^ This suggests that the relationship between the compound and the solvent molecules allow for certain interactions to occur based on different properties that were implemented within these observed systems such as inter- and intramolecular interactions. To validate this, we analyzed each of these compounds using computational quantum chemistry. The tautomers were first analyzed with 5 different DFT functionals in implicit solvents. The results of these calculations are displayed in Table 2 along with a color scheme to identify theoretical/experimental deviations that will be used throughout this work. In this scheme, a blue highlight indicates the ratios within 20% of the experimental results (which translates to a < 0.821 kcal/mol error in tautomer energy difference prediction and under the commonly accepted <1 kcal/mol definition of chemical accuracy) and the correct tautomer predicted.^37,38^ While there is not a agreed standard for how far below 1 kcal/mol one should be to ensure correct ratio prediction, we chose the 0.821 kcal/mol (20%) to ensure the ratio that is safely below the >1 kcal/mol limit even in the presence of minor experimental errors. A gray highlight indicates that either the ratio is more than 20% away from the experimental result, or the incorrect tautomer was predicted (see Table 3). The orange indicates the ratios that deviate from the experimental results entirely (i.e., both the ratio and predicted tautomer deviate from the experimental results).

**Table 1:**
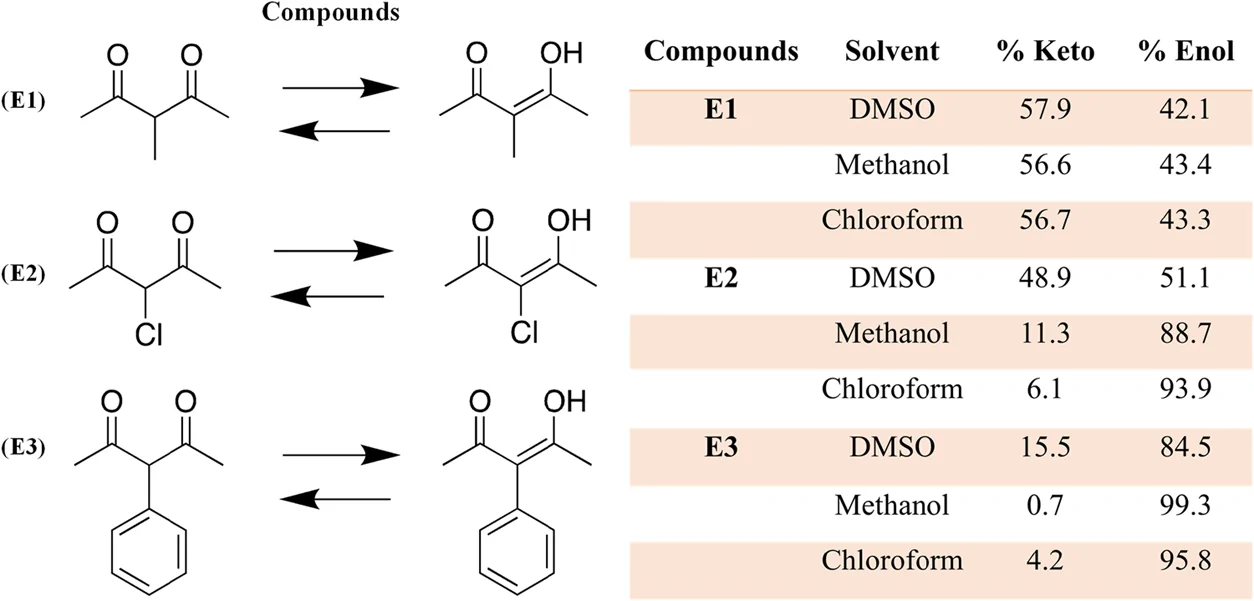
Experimental Percentage Ratios between the Keto and Enol Forms of the Corresponding Compounds

**Table 2:**
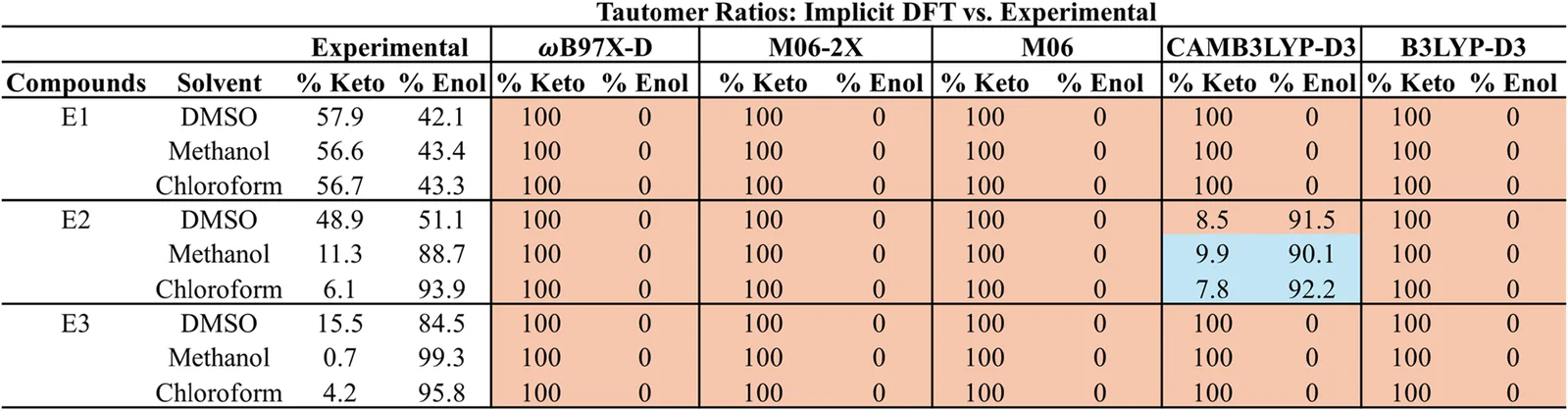
Percentage Ratios under Different DFT Methods with Implicit Solvents in Comparison to the Experimental Values

**Table 3:**
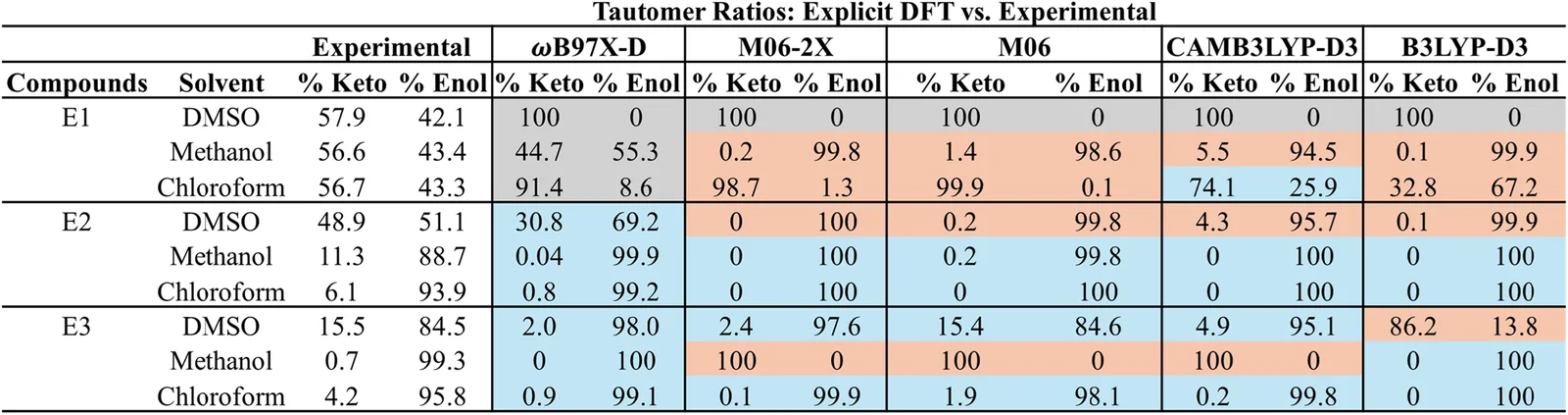
Percentage Ratios under Different DFT Methods Are Compared to the Experimental Values

These results show that, as previously seen in previous studies of other keto–enol tautomers, none of the DFT methods can consistently predict experimental tautomer ratios when using implicit solvation models alone.^39–42^ This includes a study done by several of the authors in dinitro ketone derivatives, where this was a consistent issue across a range of DFT functionals and implicit solvents.^43^ Considering the three compounds E1–E3 using purely implicit solvents, the difference in total free energies between the keto and enol tautomers are relatively large (>6 kcal/mol) making the theoretically predicted percentage ratios close to 100% in either tautomeric form (see the SI S1 for energy values). An exception is observed for compound E2 in methanol and chloroform with CAMB3LYP-D3, whose tautomeric values closely align with experimental data (>1.3 kcal/mol).

### 2.3 Evaluating Different DFT Methods under Different Explicit Solvent Conditions

The explicit solvent computational tautomer ratios are displayed in Table 3. Compared to the implicit solvent results, the performance of the DFT functionals (as defined by agreement with experimental values) varied strongly. Out of the 5 DFT functionals that were tested, the ωB97X-D functional consistently predicted tautomer ratios that were most in agreement with the experiments compared to other DFT methods. This is defined as the DFT method predicted ratio either being within 20% of the experimental ratio, predicting the same favored tautomer as the experimental results, or ideally both.

The ωB97X-D method was able to correctly either the ratio or tautomer preference in all cases and both in 6/9 cases. In contrast, the DFT functionals (CAMB3LYP-D3, B3-LYP, M06, and M06–2X) showed multiple cases of both a > 20% ratio error and incorrect tautomer prediction (orange values in Table 2). The compound E1 was the most difficult to match with ωB97X-D only able to match either the ratio or tautomer predicted but not both. This is to be expected due to the relatively equal percentage of keto and enol tautomers seen experimentally for all solvents which translates into a very small (<0.19 kcal/mol) energetic difference between the two. In previous studies of keto–enol tautomers, the predicted energetic barrier for explicit solvated systems have typically ranged from 2 to 10 kcal/mol.^44,45^ For example, in a study done by Freitag et al. regarding keto–enol tautomerization of malonaldehyde, the average relative energetic difference with zero-point vibrational energy correction included was reported to be 2.2 kcal/mol using a QM/MM computational model and B3LYP and MP2 DFT-based level of theory.^46^ For systems like E1, only ideal for the energetic barrier to be <0.19 kcal/mol as anything beyond this numerical value will result in a relatively high percentage of tautomer ratio upon converting the total free energy.

These findings are reinforced by the mean absolute error (MAE) for each DFT method shown in Figure 4. Across solvent conditions, four out of five DFT methods have high mean absolute errors in their predicted enol tautomer ratios when compared to the experimental enol tautomer findings. ωB97X-D displayed the lowest MAE overall with a value of 47% (values under all solvent conditions combined) when compared against the other DFT methods that have more than 80% MAE. Because of this, results from this method are further analyzed in the next sections of this paper.

**Figure 4:**
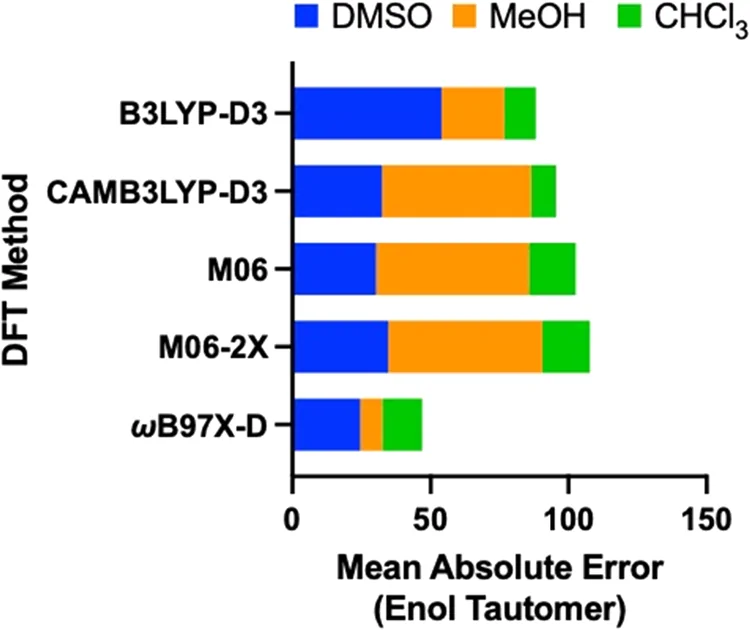
Mean absolute error between each DFT method used against the experimental % Enol tautomer ratio under different solvent conditions. Among the DFT methods, ωB97X-D had the lowest error with a 47% mean absolute error for the enol tautomer.

To explain the difference in the implicit and explicit solvent results, we checked if the solvent-induced differences in tautomer stability were due to polarity differences that resulted between tautomers. Different tautomeric forms can display major differences in their physical properties, such as dipole moments. Since the tautomeric equilibria relies intensely on the dielectric constant of the medium and the ability of solvents to form hydrogen bonds with each tautomer, more polar solvents generally favor the more polar tautomer.^47^ This is an important case since a stronger dipole moment leads to increased stability of the molecular structure in a polar solvent.^6,48^ The dipole moments (μ) of the three compounds were observed to decrease with increasing solvent polarity apart from compound E1 under methanol in which the dipole moment increased between the keto and enol tautomers as shown in Figure 5. This tells us that there is a strong preference for the keto tautomer under all different solvents; however, there is an increased stability of the enol form for compound E1 due to its stabilization through intramolecular H-bonding, resonance delocalization and a high net dipole moment of its chelated structure (Figure 5).^30,49–51^

**Figure 5:**
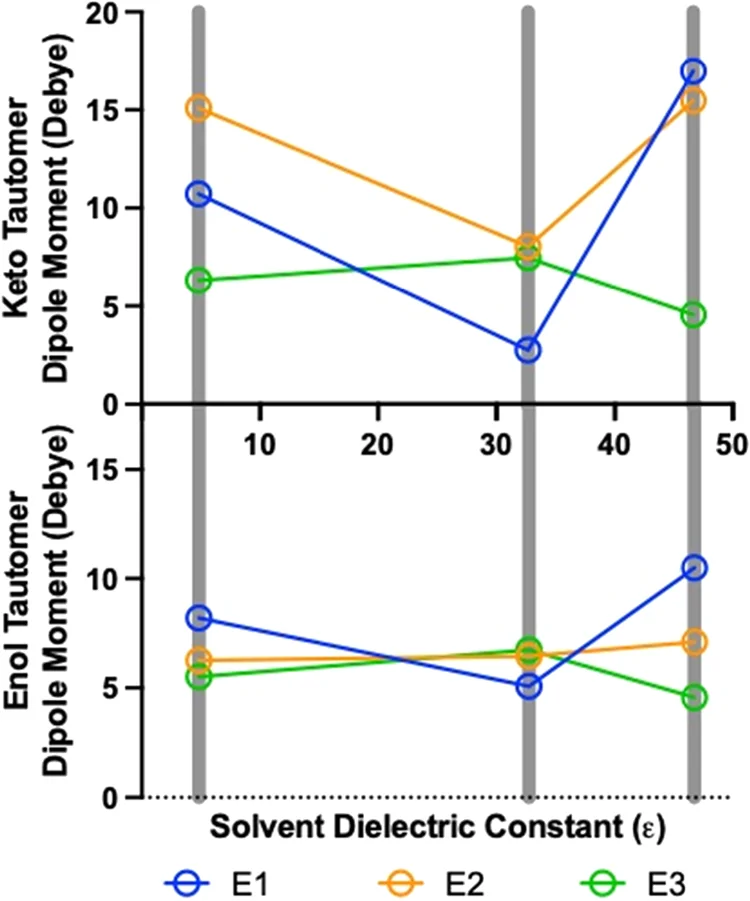
Dipole moments for each tautomer of compound E1, E2, and E3. The gray vertical lines correspond to the dielectric constant of solvents evaluated: chloroform *(*ε = 4.81), methanol (ε = 32.7), and DMSO (ε = 46.7).

### 2.4 Bridging vs Nonbridging Configurations

While explicit solvents do improve the agreement between computational predictions and experimental results, they introduce additional factors to consider compared to implicit solvents. A key challenge is that solvent shell configurations can vary strongly between tautomer configurations and potentially generate multiple energetically similar configurations for each tautomer-solvent combination.^20,52^ Addressing this requires examining how steric effects and nonbonding interactions vary between solvent-tautomer systems so that we can confirm that we are using the minimum energy configuration in our analysis.^53,54^ To check this, we thus examined how the ratios and relative energies change on the bridging versus nonbridging configurations of a hydrogen atom (via direct proton transfer in the enol tautomer of the compound) to its dicarbonyl neighbor by observing whether rotating the −OH group of the enol tautomer toward the double-bonded oxygen would create a change.

We first examined the nonbridging configuration on all three compounds to quantify the energy difference and the percentage ratios to compare its values with the experimental ones. This nonbridge configuration refers to the idea of where a direct proton transfer of a hydrogen atom is not applied in the enol tautomer, and no “bridge” was created between the dicarbonyls as seen in Scheme 1B. We then examined the bridge configuration through the rotation of a hydrogen atom from a hydroxy group in the enol form of the compound to create that “bridge” between the dicarbonyls (Scheme 1C). By rotating the atom into a position in which it can interact not only with the surrounding explicit solvent molecules, but also with the neighboring oxygen atom from a carbonyl, the effects of intermolecular and intramolecular forces affected the minimum energy configuration, thus affecting the ratios in the end. This bridged configuration reflects proximity effects, arising from interactions between a functional group and vicinal groups (including hydrogen atoms), or, in some cases, lone electron pairs. These effects involve through-space noncovalent interactions and through-bond inductive and resonance effects.^55^ While noncovalent interactions are more commonly intermolecular than intramolecular, they can either be attractive (e.g., OH···O) or repulsive (aka steric effects, e.g., O···O) depending on the fragments involved and their separation. For all three compounds across different solvent conditions, steric effects were minimal as attractive intramolecular OH···O interactions in the enol tautomer favored one tautomer over the other.^55^

**Scheme 1:**
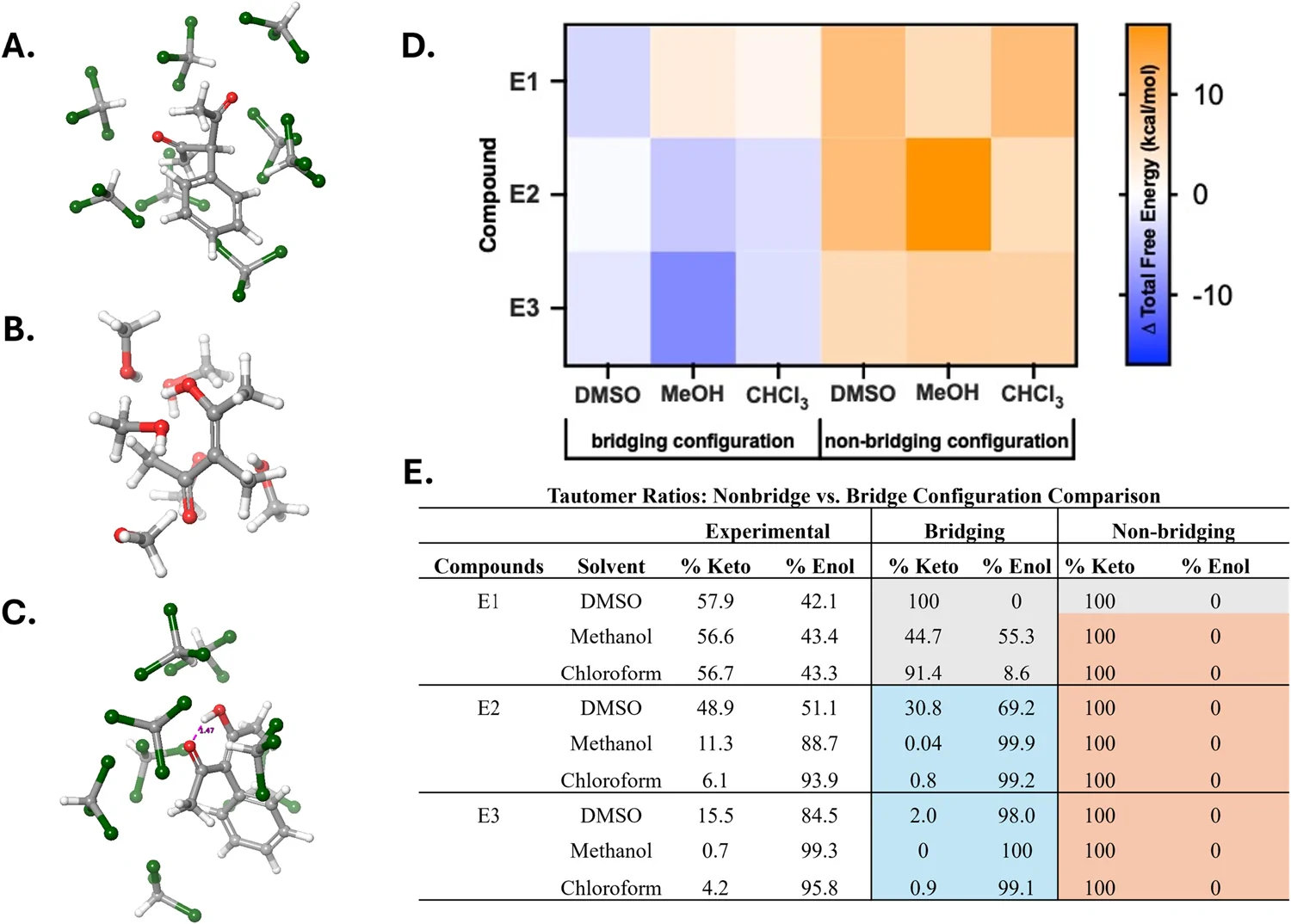
A Summary of Findings Comparing Bridge vs Nonbridge Configuration of the Hydrogen Atom Repositioning^a^

a (A) Representative structure of keto tautomer, shown in chloroform solvent, from our calculations. (B) Representative pose of “nonbridging” enol tautomer, shown in methanol solvent. (C) Representative image showing the “bridging” enol tautomer with an intramolecular hydrogen bond highlighted, shown in chloroform solvent. (D) The difference in total free energy between bridging vs. nonbridging configuration. Compounds are represented on the *X*-axis, solvents are represented on the *Y*-axis with heat map color indicating difference in total free energy (kcal/mol). (E) Calculated tautomer ratios of bridge and no-bridge effect when compared against the experimental.

The energetic results for both configurations are displayed in Scheme 1D. Upon calculating the percentage ratios between forms, Scheme 1E lists these values under the nonbridging tautomer category. The difference in total free energies for the nonbridging tautomers shows that in which a good majority of the three compounds displayed a preference of the keto tautomer over the enol one by more than 5 kcal/mol. The comparison between the experimental and the nonbridging ratios showed a drastic deviation where the theoretical methods predict 100% keto tautomer. Between these two data sets, the bridged confirmations predict that the majority of the compounds favored by the enol tautomer. This was further validated when the energy values were converted into their respective percentage ratios as indicated in Scheme 1E under the bridging configuration category of the data table.

Despite preferring the bridging configuration, the resulting values for compound E1 do not fully match the experimental values as discussed in the previous section. This indicates that, other than the hydrogen-bonding interaction between the compound and its surrounding explicit solvent molecules, other interactions, such as intramolecular interactions, can also play a role in the shifting between keto and enol ratios. Another possibility is whether the deployed DFT method and basis set could have overestimated the result upon calculation/simulation.

### 2.5 Computational Evaluation of Additional Tautomers Compounds and Their Trends

Substituent effects are well-known to play a significant role in the electronic energies by causing shifts in the electron density through electron withdrawal or donation by different substituents.^55^ To evaluate how substituent groups affect keto–enol tautomer ratios for the E1–E3 compounds, we computationally examined two alternative substituents for the E3 keto–enol tautomer, tautomer pairs C1 and C2 shown in Scheme 2. Compounds C1 and C2 have a methoxy and chlorine atom substituent on the phenyl ring respectively, thus different electron withdrawing characteristics compared to E3. According to the substituent priority rules in organic chemistry, one would expect C2 to be more electron withdrawing than E3 and C1 less resulting in increased enol favoritism from C1→E3→C2. Conceptually, we envision two aspects of structural influence on tautomer distribution.^56,57^ The first involves electronic stabilization and/or destabilization of any charge to build up during the transition state for enol-keto tautomerization. The second involves specific structural stabilization of one tautomeric form. As these effects are electronic in nature, we anticipate the ability to computationally identify changes from structurally distant changes. For example, in molecules C1 and C2 the introduction of a structurally distant weakly inductively withdrawing substituent (para-Cl; Hammett constant (σ_p_ = +0.23)) contrasts the introduction of a distant donating substituent (para-OMe; Hammett constant (σ_p_= −0.27)). These changes would be anticipated to impact the transition state for tautomerization and additionally may provide direct stabilization and/or destabilization of the enol form of the compound in which the distant functional group is placed in conjugation (Scheme 2).

**Scheme 2:**
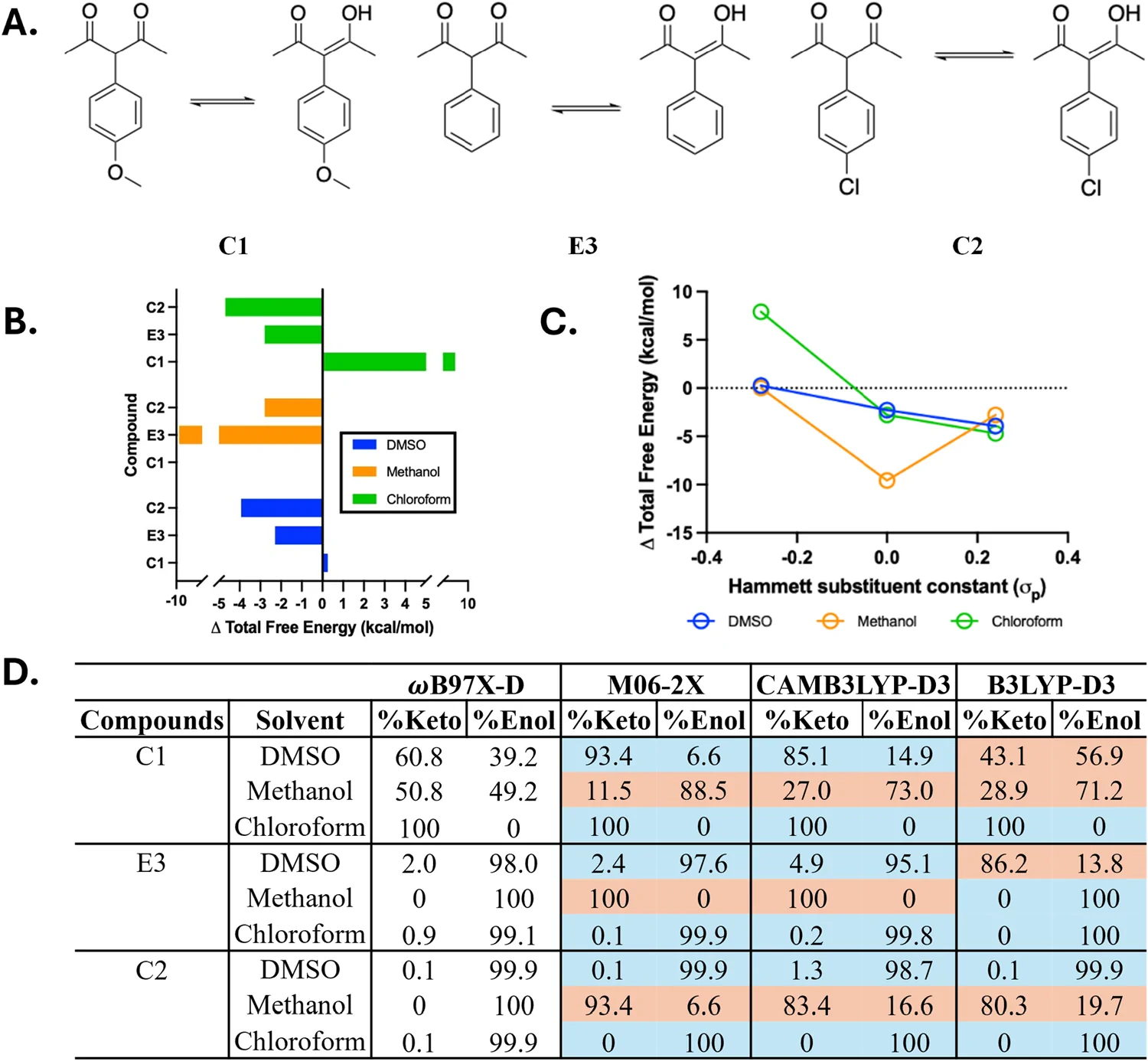
Characterizing Remote Electronic Effects Impacting Predicted Tautomer Distribution^a^

a (A) Compound C1 is predicted to favor the keto form while compound C2 favors the enol form due to their differing substituents. A summary of all theoretical findings to verify a trend based on compounds C1, E3, and C2. (B) The difference in total free energy between tautomers (ωB97X-D) under different solvent conditions. (C) Correlation of total free energy difference with Hammett substituent constant demonstrating the role of remote electronic contributions to tautomerization. (D) DFT comparison to determine the tautomeric ratios with ωB97X-D ratios serving as the standard.

These basic organic chemistry assumptions of distant structural effects on chemical reactivity are supported by the ωB97X-D predictions. As shown in Scheme 2B, the energetic values predict that compound C1 generally favors the keto form while C2 strongly favors the enol tautomer under all solvent conditions. The baseline compound E3 also strongly favored the enol especially with a methanol solvent, the only solvent where C2 is less enol-favored than E3. This observation aligns with the well-established stabilization mechanisms of 1,3-diketone enols, namely the formation of a six-membered intramolecular hydrogen bond (O–H···O) and extended π-conjugation involving both carbonyl groups. This correlates with compounds E3/C2 having substituents with an electron-withdrawing character and C1’s substituent having a net electron-donating character.

This is further evidenced in Scheme 2D, where the percentage ratios between the keto and enol tautomers are presented. For ωB97X-D, compounds E3 and C2 display near 100% enol preference as expected while C1 favors its keto form by varying degrees across the 3 solvent types. Compared to ωB97X-D, other DFT methods were also explored to show clear differences in predicted ratios with B3LYP-D3 showing the highest variance as seen in Scheme 2D. Under the B3LYP-D3 method, compound C1 exhibited opposite behavior in DMSO and methanol conditions, while E3 showed a similar reversal only in DMSO. In contrast, C2 displayed consistent behavior exclusively in methanol. A similar pattern was observed with M06–2X and CAMB3LYP-D3 methods in methanol, where C1 favored the enol tautomer, while the other two compounds predominantly favored the keto form with populations ranging from approximately 80–100%. When compared to the ωB97X-D results, these findings highlight significant variations between the other computational methods.

However, the view on resonance effects should be analyzed with caution, as steric factors may significantly perturb conjugation by twisting the aromatic ring out of coplanarity in certain systems. For compounds C1–C2, the phenyl group is expected to adopt a near-perpendicular orientation relative to the plane of the intramolecular hydrogen bond, thereby limiting effective π-overlap and diminishing resonance delocalization. In contrast, compound E3 may adopt a more torsional angle of approximately 45°, likely due to reduced steric congestion, which could permit a relatively greater electronic communication between the aromatic substituent and the enol framework. Nevertheless, the overall influence of substituent resonance effects on the enol moiety is expected to remain minimal, as the reduced planarity in these systems substantially diminishes conjugative interactions. This behavior is further supported from the ^1^H NMR chemical shifts of -OMe and -Cl substituted compounds, which exhibited nearly identical resonance positions.^58^

We then examined the electron donating/withdrawing characteristics for compounds C3 and C4 to evaluate DFT integration of direct heteroatom donating effects on tautomerization. It is expected for compound C4 to be more enol-favoring than compound C3 since the acetoxy functional group is a better electron withdrawing group (weaker donating group) compared to C3. The theoretical results support this expectation as shown in Scheme 3B in which the total free energy difference under DMSO and methanol indicates that compound C4 is more enol-favoring than compound C3. We were unable to find stable minimum energy structure for these compounds in chloroform, so the solvent was excluded from the analysis.

**Scheme 3:**
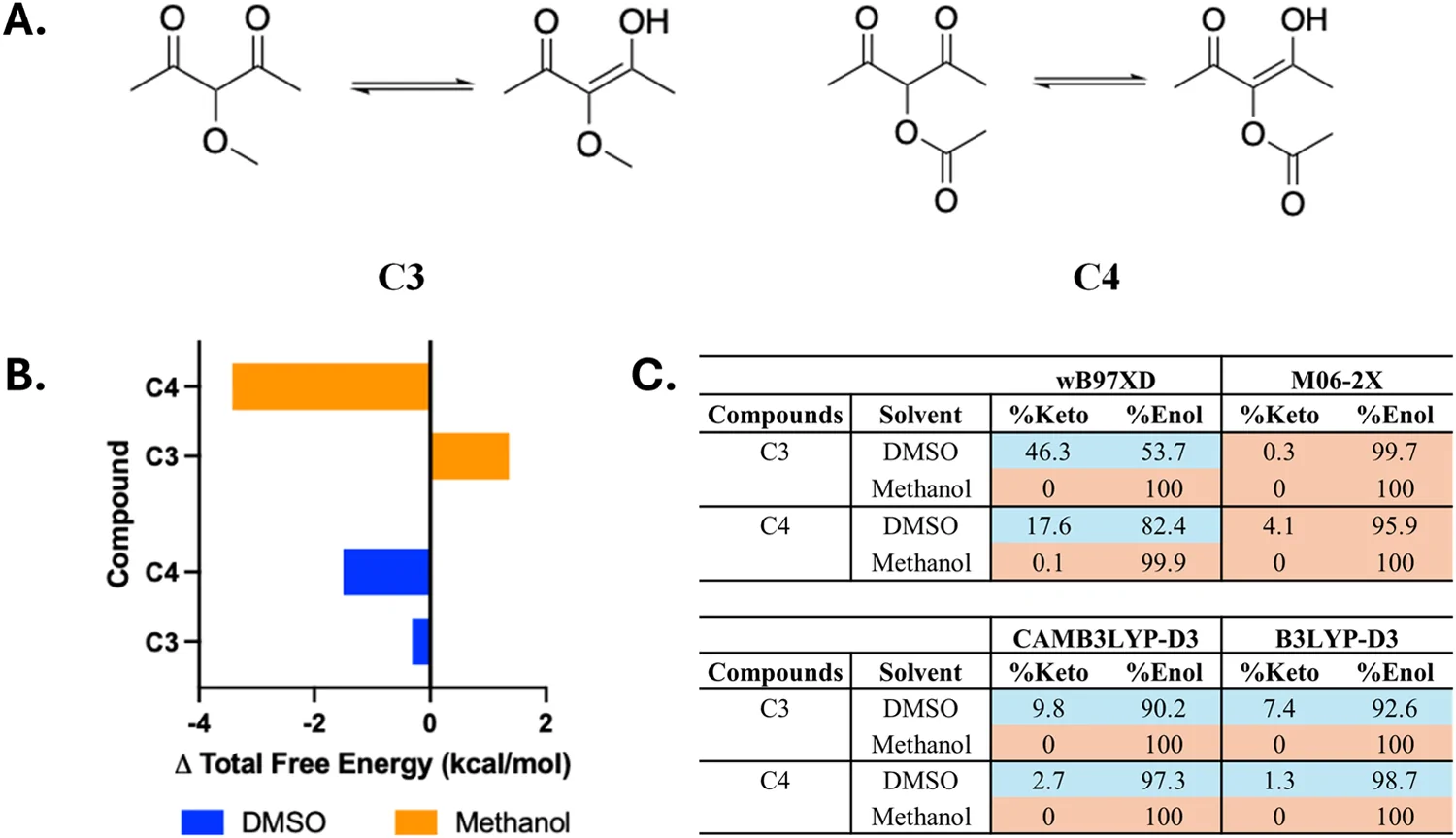
A Trend Highlighting Compounds from Increasing Enol-Favoring^a^

a (A) Compound C3 has a methoxy functional group while compound C4 has an acetoxy (OAc) functional group. A summary of all theoretical findings to verify a trend based on compounds C3 and C4. (B) The difference in total free energy under different solvent conditions. (C) A DFT comparison to determine the tautomeric ratios.

Although compound C3 with a methoxy substituent is keto-favoring under methanol, it still follows the direction in which compound C4 is favoring the enol tautomer more than compound 3 due to resonance effect. By using ωB97X-D as the standard DFT method, Scheme 3C validates this finding with C3 favoring 54% of the enol tautomer, but C4 overtakes this as it favors 82% of the enol in DMSO. With DMSO acting as a base reagent, the description in which the compounds undergo a reaction under a basic solution solidifies the idea that compound C4 will likely experience more resonance due to the distribution of electrons from the oxygen atom of the acetoxy functional group toward the anion charge on the α carbon and toward the rest of the group. This results in a strong predicted preference for the enol tautomer.

A DFT comparison was also done for this analysis of the trend as shown in Scheme 3C. We were unable to find stable minimum energy structure for these compounds in chloroform, so the solvent was excluded from the analysis. While ωB97X-D, CAMB3LYP-D3, and B3LYP-D3 follow the expected trend in which the compound with OAc as its substituent being the most enol-favoring, ωB97X-D continue to prove to be the most effective DFT method that can predict the percentage ratios between tautomeric forms as mentioned previously, creating a 28% difference between the two. The percentage ratios under M06–2X as its DFT method, on the other hand, created the opposite effect in which C3 exhibited a preference for the enol tautomer under DMSO more than C4. This was indicated by C3′s propensity to choose 100% of the enol form, in contrast to C4’s 95%. Furthermore, the ratios under methanol conditions delivered unforeseen findings in which both compounds preferred the enol tautomer 100%, which tells us that both systems are too polar. This creates a form of challenge as it is hard to distinguish whether compound C4 is more enol-favoring than C3.

## 3 Conclusion

In this paper, we examined the impact of different substituents, solvent conditions, DFT functional choice, and reorientation on the keto–enol equilibrium ratios of several dicarbonyl derivatives. Based on the results and observations reported, factors such as different DFT methods, the presence of explicit solvents, substituent withdrawing character, and the molecule’s orientation affected the equilibrium ratio between the keto and enol forms. Among the DFT methods applied, QM/MM using the ωB97X-D functional and an explicit solvent shell gave the most accurate values in terms of percentage ratios when compared to experimental data. Although reproducing the experimental ratios for compound E1 proved particularly challenging – likely due to inherent methodological limitations such as functional-dependent approximations – the overall results demonstrate that QM/MM calculations employing the ωB97X-D functional or a similar quality range separated functional with explicit solvent provide consistent and reliable predictions, successfully replicating experimental tautomeric ratios across a wide range of solvents in most cases. Analyzing both the bridging and nonbridging configurations also suggests that a bridging configuration is the preferred minimum energy structure for these systems. Furthermore, applying the current computational methods to the second phase of our analysis, which involved a new set of compounds (C1–C4), enabled us to examine how different substituents influence tautomeric ratios. A close comparison of the dicarbonyl derivatives with methoxy and chlorine substituents (compounds C1 and C2, respectively) revealed that the chlorine-substituted aromatic ring (C2) exhibited a significantly stronger electron-withdrawing character than its methoxy counterpart (C1). This trend was also observed in compounds C3 and C4, where the acetoxy-substituted molecule (C4) showed greater electron-withdrawing ability to the methoxy-substituted analog (C3).

Future theoretical analyses and experiments will need to be made by considering other potential factors such as basis set effects, the effects of pH on a given solvation model, and the role of temperature in these systems. Considering the cost of functionals like ωB97X-D, alternative lower-cost electronic structure methods based on machine learning are also worth considering in future studies.

## 4 Materials and Methods

### 4.1 Computational Methods

Geometry optimizations for each compound were done using implicit solvents (methanol, DMSO, and chloroform) using Schrodinger’s Jaguar Software.^59^ We initially optimized the tautomers in implicit solvents using 5 density functional theory methods: B3LYP-D3, M06, M06–2X, CAMB3LYP-D3, and ωB97X-D using the 6–311G++** basis set. We primarily aimed to examine representative DFT functionals (particularly the M06 and B3LYP families) to identify those capable of accurately predicting the tautomeric ratio at a reasonable computational cost so a reasonably large basis set with both diffuse and polarization functions was chosen. We choose two representative Minnesota functionals (M06 and M06–2X) in implicit solvent calculations, focusing on those with a sufficient fraction of Hartree–Fock exchange (27 and 54% of HF exchange). For the remaining functionals, B3LYP-D3 was as a baseline, with CAMB3LYP-D3 chosen to represent functionals with long-range corrections to B3LYP which were expected to be important for modeling explicit solvent interactions. ωB97X-D was chosen as a representative non-B3LYP-based range-separated hybrid functional. Afterward, several keto–enol molecular models were created using Schrodinger’s Jaguar Software with explicit solvent molecules such as methanol, DMSO, and chloroform surrounding the observed compounds.^59^ These systems consider both the keto and enol tautomeric forms, thus creating 18 solvated systems in total. Geometry optimizations for the explicit solvent models were performed using Quantum Mechanic/Molecular Mechanics (QM/MM) with the entire tautomer and the closest solvent molecule in the Quantum Mechanical region and the rest of the solvent molecules treated using molecular mechanics. Due to computational cost considerations, the optimizations were carried out using B3LYP-D3 and 6–31G++** as the DFT method and basis set, respectively. To improve the accuracy of the final explicit tautomer ratios, the single point energy QM/MM calculations were done in Gaussian 16 using the same 5 DFT methods as the implicit solvent models and the larger/more accurate Def2-QZVP basis set to acquire the predicted thermochemical properties and tautomer ratios that can be compared to the experimental values (See Experimental Methods).^60^ All thermochemical properties were set at 298.15 K and 1 atm and in addition to the explicit solvents, the conductor-like polarizable continuum model (CPCM) model was used. The solvents that were used for CPCM are the same as the explicit solvents that were constructed that surrounded the system.

After acquiring the Gibbs free energies of all seven compounds under different solvent conditions, the tautomeric equilibrium constants between the keto and enol forms were calculated using the tautomeric equilibrium constants (*K*_eq_) as follows:^6^

1 $$K_{\mathrm{eq}}=\exp\left(-\frac{\Delta G}{\mathrm{RT}}\right)$$

where *R* denotes the gas constant (*R* = 0.001985 kcal mol^–1^ K^–1^), *T* is the standard temperature of 298.15 K, and Δ*G* represents the difference between the Gibbs free energies of the keto and enol tautomers. The calculated equilibrium constants for each compound are available in the SI. In addition, the percentage ratios between the keto and enol forms were determined by using the mole fractions of the two tautomers:^6^

2 $$\%\mathrm{Keto}=\left(\frac{K_{\mathrm{eq}}}{1+K_{\mathrm{eq}}}\right)\times 100$$

3 $$\%\mathrm{Enol}=\left(\frac{1}{1+K_{\mathrm{eq}}}\right)\times 100$$

Owing to the greater thermodynamic stability of the keto tautomer relative to the enol form, eq 2 is employed to determine the percent ratio of the keto species.^6^

### 4.2 Experimental Methods

Experimental determination of equilibrium enol-keto ratios at ambient temperature was determined by NMR spectroscopy in d6-dimethylsulfoxide, d4-methanol and d1-chloroform. All chemicals and deuterated solvents were purchased from commercial vendors and were used without further purification (see SI for vendor and purity information). ^1^H NMR spectra were recorded using a Bruker Avance Neo spectrometer (400 MHz). Chemical shifts are reported in parts per million (ppm) and were calibrated according to residual protonated solvent or TMS.

## Supplementary Material



## Notes

Additional theoretical and experimental data; and molecular structures of the analyzed systems (PDF)
