# Supplementary material for: Linking Enol–Keto Tautomerization Ratios in β‑Diketones to Their Physical Properties Using Computational Quantum Chemistry
Source: ACS Omega. 2026 Aug 13;11(33):49682–91. doi: 10.1021/acsomega.6c03902 (PMC13520028; doi:10.1021/acsomega.6c03902)

Supporting Information

Linking Enol-Keto Tautomerization Ratios in Beta-Diketones to Physical Properties using Computational Quantum Chemistry

Julia Gabriel, Jesse Na, Brian Guillermo, Lark J. Perez*, and Erik P. Hoy*

Rowan University

201 Mullica Hill Road, Glassboro, NJ 08028

*Emails: perezla@rowan.edu; hoy@rowan.edu

Supporting Information

	Total Free Energy Difference (in kcal/mol)						
	Compounds	Solvents	M06	M06-2X	CAMB3LYP-D3	B3LYP-D3	wB97X-D
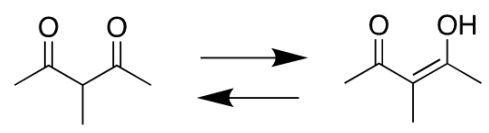	E1	DMSO	9.50	8.90	45.3	9.65	11.71
		Methanol	9.58	9.49	45.3	9.63	11.68
		Chloroform	9.19	8.63	45.7	9.69	11.16
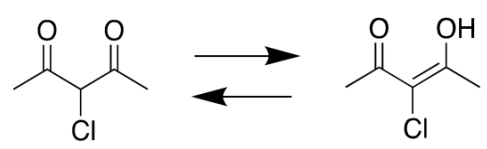	E2	DMSO	7.45	6.02	-1.41	7.51	7.72
		Methanol	6.91	6.71	-1.31	7.33	7.68
		Chloroform	7.46	6.54	-1.46	7.78	7.53
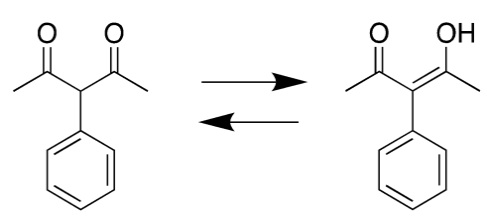	E3	DMSO	7.56	6.40	35.5	7.16	8.44
		Methanol	7.46	6.39	35.5	7.15	8.40
		Chloroform	7.30	6.24	35.3	6.55	8.19

Table S1. *Total free energy difference across DFT methods using implicit solvation model in the units of kcal/mol.*

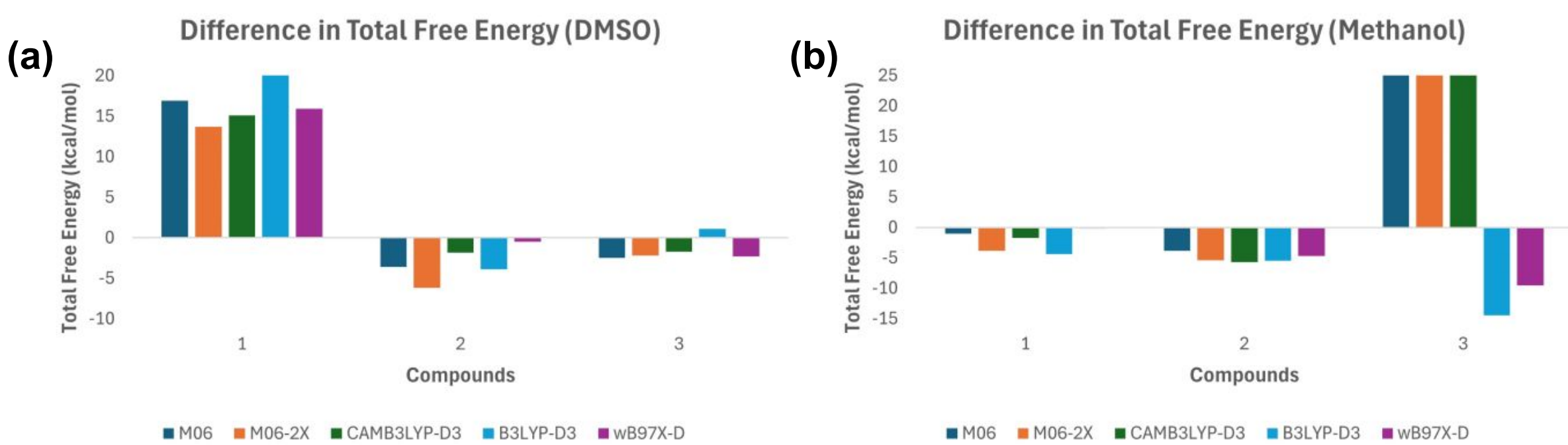

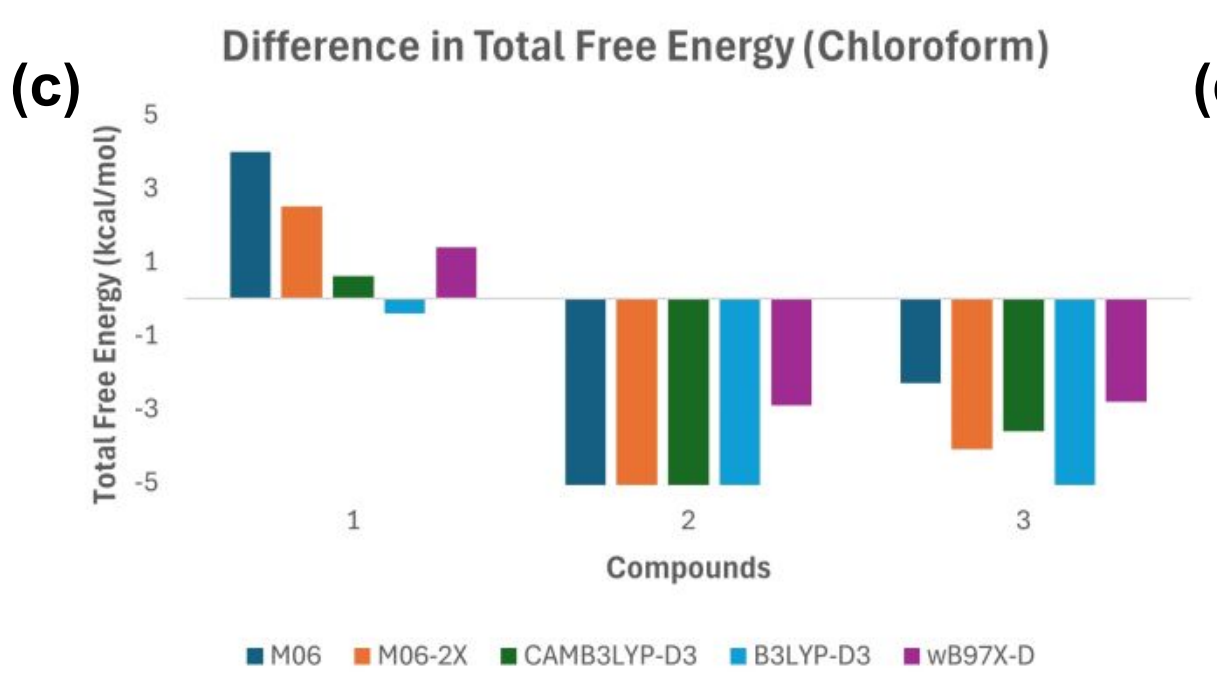

(d)

Total Free Energy Difference (in kcal/mol)						
Compounds	Solvents	M06	M06-2X	CAMB3LYP-D3	B3LYP-D3	wB97X-D
1	DMSO	16.9	13.7	15.1	75.2	15.9
	Methanol	-1.0	-3.8	-1.7	-4.4	-0.1
	Chloroform	4.0	2.5	0.62	-0.4	1.4
2	DMSO	-3.6	-6.2	-1.84	-3.9	-0.5
	Methanol	-3.8	-5.4	-5.7	-5.5	-4.7
	Chloroform	-6.3	-9.0	-6.2	-10.1	-2.9
3	DMSO	-2.5	-2.2	-1.75	1.1	-2.3
	Methanol	32.5	30.9	35.1	-14.5	-9.6
	Chloroform	-2.3	-4.1	-3.6	-6.9	-2.8

Figure S1. *Tabulated bar graphs of the total free energy difference across DFT methods in DMSO (a) methanol (b), chloroform (c) using explicit solvation model. The full numerical results are included in (d).*

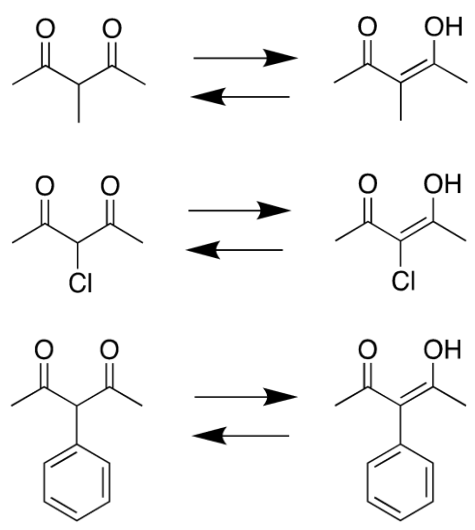

		Dipole Moments		
Compounds	Tautomers	Chloroform (e = 4.81)	Methanol (e = 32.7)	DMSO (e = 46.7)
E1	Keto	10.719	2.763	16.981
	Enol	8.197	5.075	10.495
E2	Keto	15.097	8.048	15.476
	Enol	6.259	6.463	7.106
E3	Keto	6.324	7.475	4.558
	Enol	5.513	6.741	4.562

Table S2. *The dipole moments for each compound (from Series 1) and dielectric constants for each solvent.*

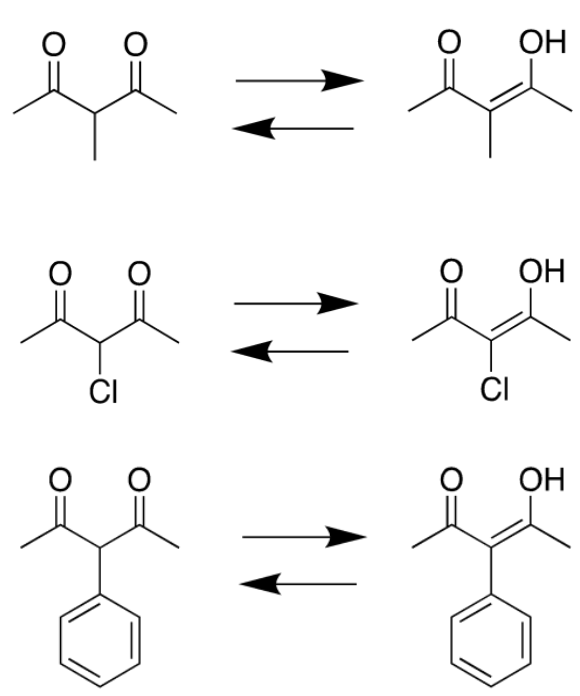

		Total Free Energy Difference (in kcal/mol)		
Compounds	Tautomer form	DMSO	Methanol	Chloroform
E1	Keto	-4257.85858	-1542.55441	-11740.04272
	Enol	-4257.84210	-1542.54527	-11740.02601
	ΔGketo-enol (kcal/mol)	10.342	5.737	10.482
E2	Keto	-4678.39680	-1615.76020	-12160.37250
	Enol	-4678.38030	-1615.73520	-12160.36357
	ΔGketo-enol (kcal/mol)	10.354	16.990	5.599
E3	Keto	-4449.54936	-1502.70871	-11931.74192
	Enol	-4449.53988	-1502.69657	-11931.73041
	ΔGketo-enol (kcal/mol)	5.947	7.620	7.226

Table S3. *The total free energy difference (in kcal/mol) between the keto and enol forms for all three compounds under different solvent conditions using the non-bridging effect.*

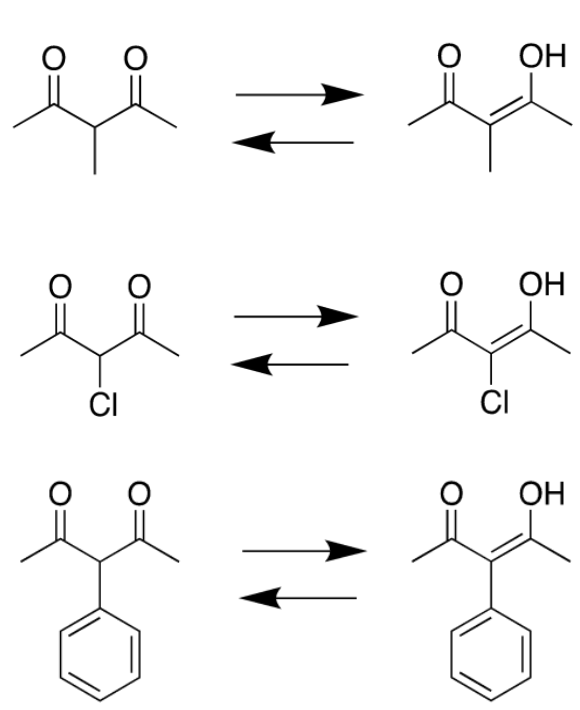

		Total Free Energy Difference (in kcal/mol)		
Compounds	Tautomer form	DMSO	Methanol	Chloroform
E1	Keto	-4257.78398	-1542.50119	-11740.01557
	Enol	-4257.78940	-1542.49635	-11740.01333
	ΔGketo-enol (kcal/mol)	-3.402	3.041	1.406
E2	Keto	-4124.92049	-1615.59352	-12160.37694
	Enol	-4124.92126	-1615.60099	-12160.38148
	ΔGketo-enol (kcal/mol)	-0.481	-4.685	-2.846
E3	Keto	-4449.55114	-1734.14465	-11931.74048
	Enol	-4449.55479	-1734.15992	-11931.74492
	ΔGketo-enol (kcal/mol)	-2.288	-9.579	-2.790

Table S4. *The total free energy difference (in kcal/mol) between the keto and enol forms for all three compounds under different solvent conditions using the bridging effect. The values for compounds 1 indicate inconsistencies in contrast to the experimental results which further supports the method of repositioning/reorienting the hydrogen atom.*

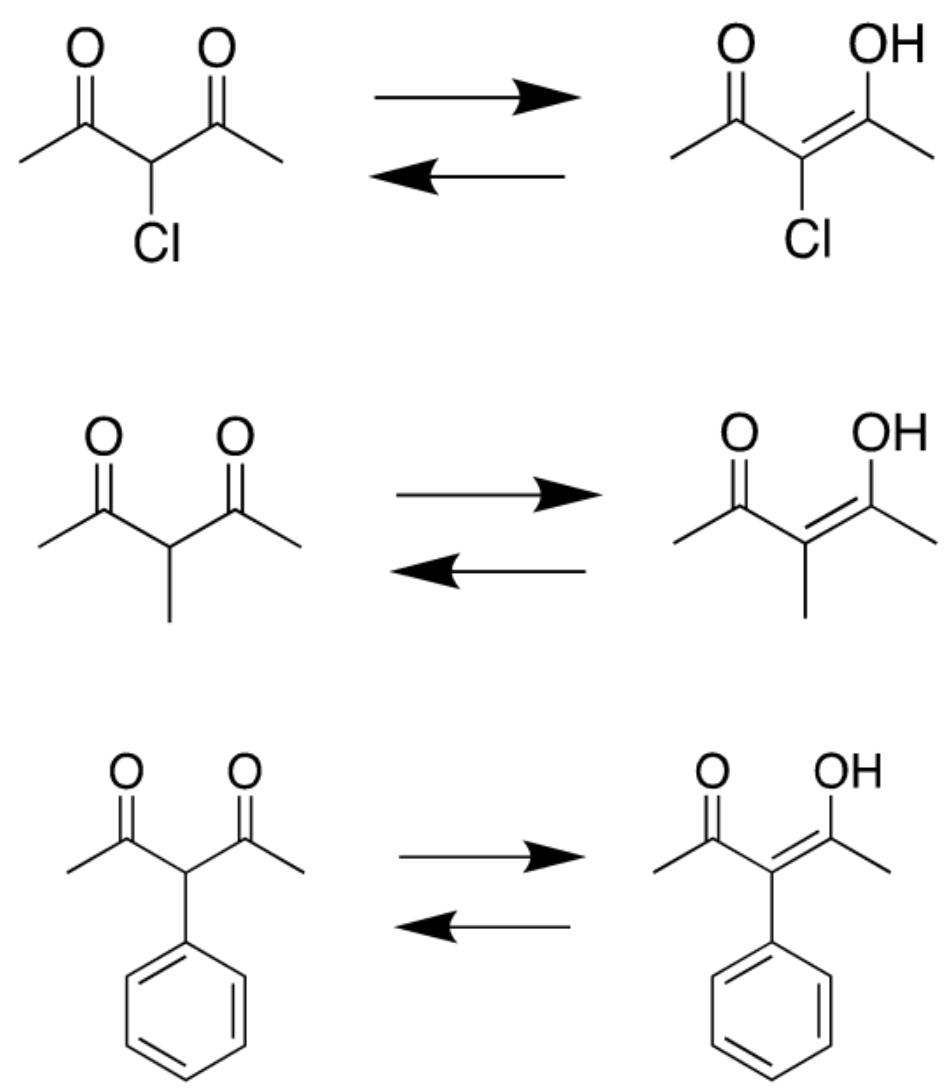

Compounds	Solvent	Keq
E1	DMSO	5.32634E+11
	Methanol	0.806872622
	Chloroform	10.64993
E2	DMSO	0.444393
	Methanol	0.000368
	Chloroform	0.008102
E3	DMSO	0.020872
	Methanol	9.33283E-08
	Chloroform	0.008967

Table S5. *The tautomeric equilibrium constants of all three compounds under different solvation conditions.*

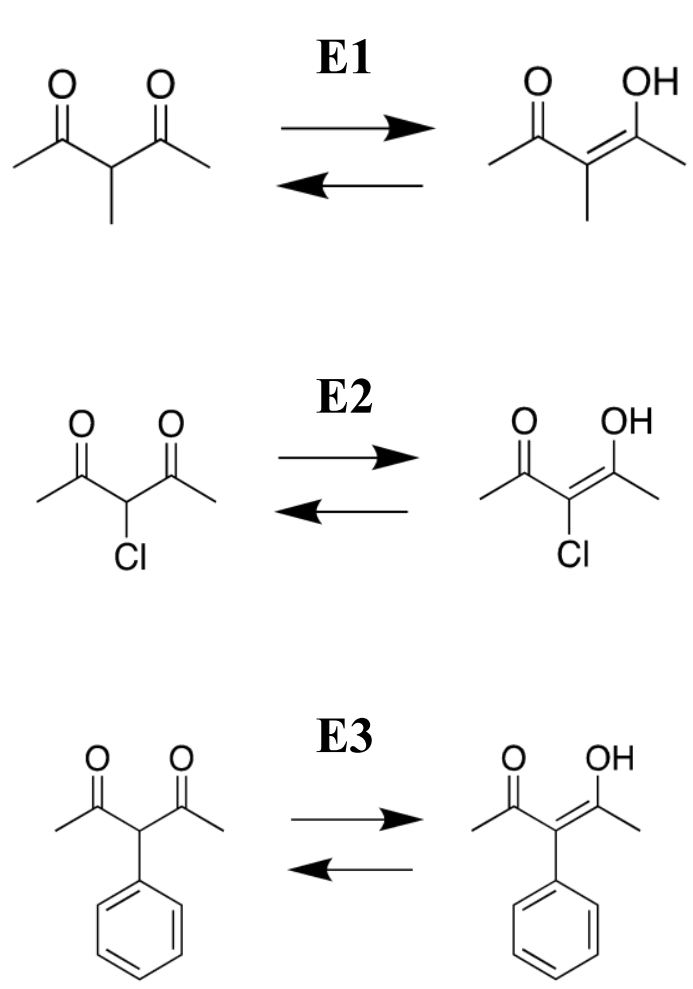

Thermodynamic Properties

A.

Compounds	Tautomer form	Gibbs Free Energy (kcal/mol)	Enthalpy (kcal/mol)	Entropy (kcal/mol)
E1	Keto	-24.352	8.646	110.677
	Enol	-24.256	8.479	109.795
E2	Keto	-24.757	8.626	111.967
	Enol	-24.556	8.620	111.272
E3	Keto	-27.599	11.082	129.736
	Enol	-27.087	10.676	126.656

B.

Compounds	Tautomer form	Gibbs Free Energy (kcal/mol)	Enthalpy (kcal/mol)	Entropy (kcal/mol)
E1	Keto	-26.736	10.676	125.479
	Enol	-26.601	10.613	124.815
E2	Keto	-26.980	10.546	125.863
	Enol	-25.722	9.793	119.118
E3	Keto	-29.046	12.590	139.649
	Enol	-28.451	12.166	136.23

C.

Compounds	Tautomer form	Gibbs Free Energy (kcal/mol)	Enthalpy (kcal/mol)	Entropy (kcal/mol)
E1	Keto	-26.913	10.163	124.354
	Enol	-26.276	9.812	121.042
E2	Keto	-26.694	9.645	121.881
	Enol	-26.517	9.624	121.216
E3	Keto	-28.947	11.862	136.876
	Enol	-28.995	11.787	136.783

Table S6. *Thermodynamic properties assuming an ideal gas under different solvent conditions for compounds E1-E3.* ***A)*** *Methanol results.* ***B)*** *DMSO results.* ***C)*** *Chloroform results.*

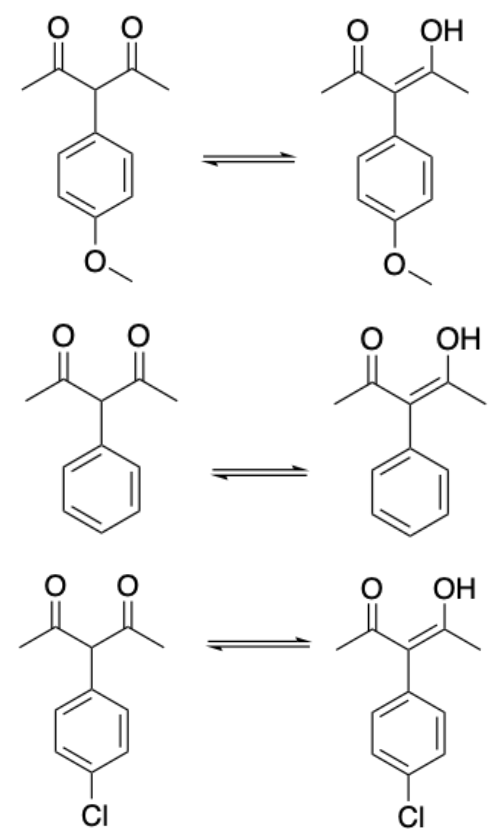

Total Free Energy Difference (in kcal/mol)				
**Compounds**	**Tautomer form**	**DMSO**	**Methanol**	**Chloroform**
C1	Keto	-4564.06838	-1501.41513	-10626.86802
	Enol	-4564.06797	-1501.41511	-10626.85543
	ΔGketo-enol (kcal/mol)	0.257	0.0151	7.898
E3	Keto	-4449.55110	-1734.14465	-11931.74050
	Enol	-4449.55480	-1734.15992	-11931.74492
	ΔGketo-enol (kcal/mol)	-2.322	-9.579	-2.774
C2	Keto	-4909.18662	-1846.53059	-10971.98890
	Enol	-4909.19288	-1846.54114	-10971.99636
	ΔGketo-enol (kcal/mol)	-3.928	-6.617	-4.681

Table S7. *The total free energy difference (in kcal/mol) between the keto and enol forms of the novel compounds under all solvent conditions.*

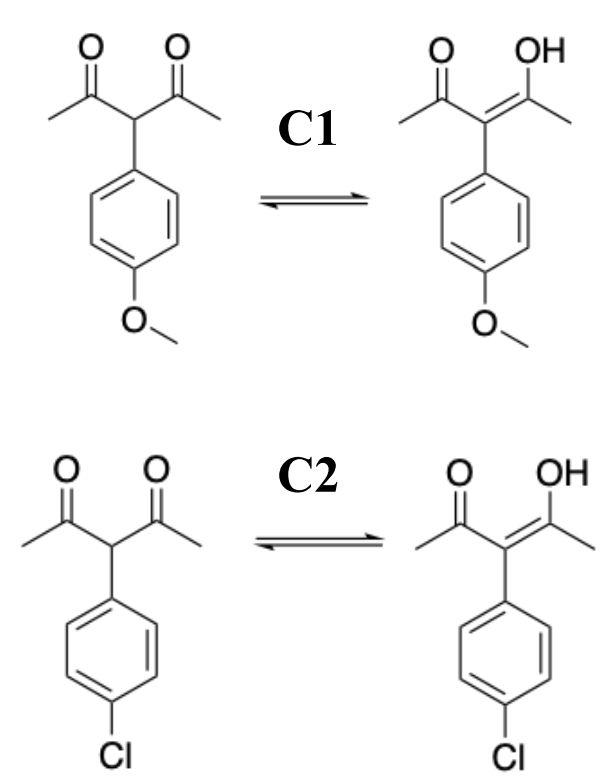

Thermodynamic Properties

A.

Compounds	Tautomer form	Gibbs Free Energy (kcal/mol)	Enthalpy (kcal/mol)	Entropy (kcal/mol)
C1	Keto	-32.387	15.092	159.246
	Enol	-32.665	15.285	160.826
C2	Keto	-32.021	14.528	156.126
	Enol	-31.805	14.389	154.934

B.

Compounds	Tautomer form	Gibbs Free Energy (kcal/mol)	Enthalpy (kcal/mol)	Entropy (kcal/mol)
C1	Keto	-30.604	14.122	150.011
	Enol	-30.402	13.892	148.562
C2	Keto	-30.058	13.28	145.356
	Enol	-29.517	12.997	142.593

C.

Compounds	Tautomer form	Gibbs Free Energy (kcal/mol)	Enthalpy (kcal/mol)	Entropy (kcal/mol)
C1	Keto	-35.354	17.101	175.935
	Enol	-30.439	13.315	146.752
C2	Keto	-29.912	12.692	142.894
	Enol	-29.796	12.531	141.966

Table S8. *Thermodynamic properties assuming an ideal gas under different solvent conditions for compounds C1-C2.* ***A)*** *Methanol results.* ***B)*** *DMSO results.* ***C)*** *Chloroform results.*

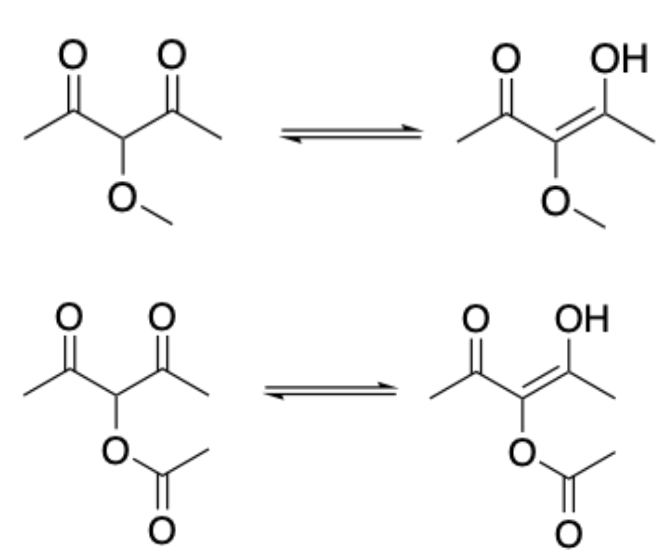

Total Free Energy Difference (in kcal/mol)			
**Compounds**	**Tautomer form**	**DMSO**	**Methanol**
C3	Keto	-4333.06235	-1849.21127
	Enol	-4333.06284	-1849.20910
	ΔGketo-enol (kcal/mol)	-0.307	1.364
C4	Keto	-4446.43149	-1962.56877
	Enol	-4446.43387	-1962.57421
	ΔGketo-enol (kcal/mol)	-1.495	-3.414

Table S9. *The total free energy difference (in kcal/mol) between the keto and enol forms of the novel compounds 6 and 7 under DMSO and methanol.*

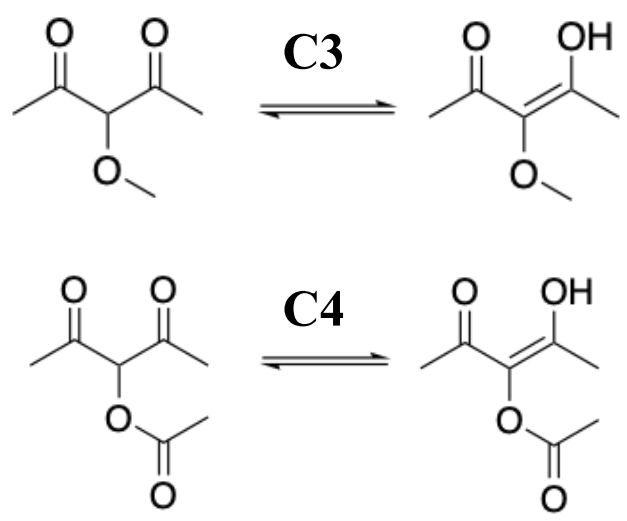

Thermodynamic Properties

A.

Compounds	Tautomer form	Gibbs Free Energy (kcal/mol)	Enthalpy (kcal/mol)	Entropy (kcal/mol)
C3	Keto	-25.766	9.748	119.112
	Enol	-24.987	9.388	115.295
C4	Keto	-27.154	10.849	127.463
	Enol	-26.522	10.505	124.19

B.

Compounds	Tautomer form	Gibbs Free Energy (kcal/mol)	Enthalpy (kcal/mol)	Entropy (kcal/mol)
C3	Keto	-26.785	11.073	126.976
	Enol	-26.310	10.683	124.075
C4	Keto	-28.152	12.092	134.98
	Enol	-28.344	12.158	135.846

Table S10. *Thermodynamic properties assuming an ideal gas under different solvent conditions for compounds C3-C4.* ***A)*** *Methanol results.* ***B)*** *DMSO results.* ***C)*** *Chloroform results.*

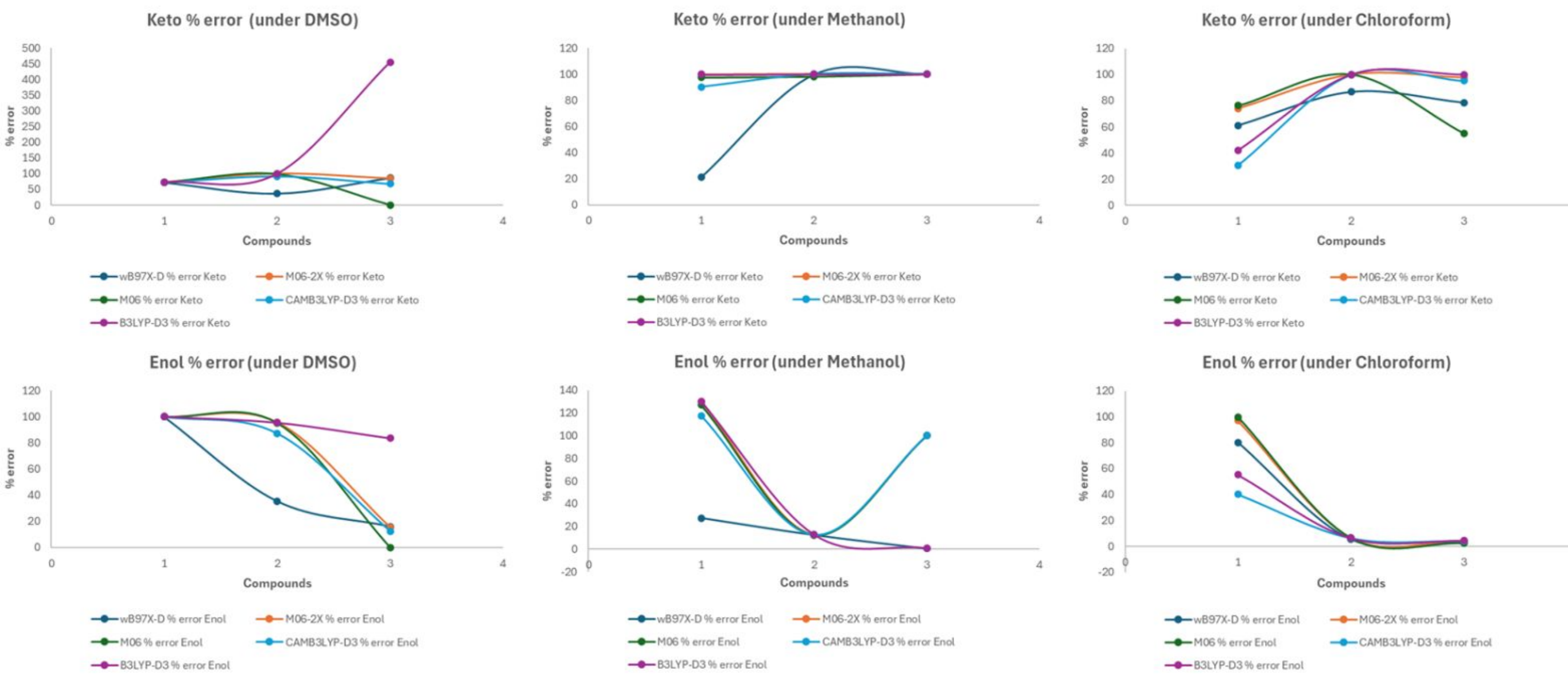

Figure S2. *Percent errors of each DFT method between the keto and enol tautomers under different solvent conditions.*

Table S11. Table of commercial suppliers and purity for chemicals and solvents.

Compound	***Supplier***	***Supplier Number***	***Purity***
3-phenyl-2,4-pentanedione	Tokyo Chemical Industry Co. Ltd.	P1297	≥98.0%
3-methyl-2,4-pentanedione	Ambeed, Inc.	A653322	≥97%
3-chloroacetylacetone	Tokyo Chemical Industry Co. Ltd.	C1277	≥95.0%
d4-methanol	VWR International Ltd.	BDH0913	99.8% D
d1-chloroform	Cambridge Isotope Labs Inc.	DLM-7	99.8% D
d6-dimethylsulfoxide	Cambridge Isotope Labs Inc.	DLM-10	99.9% D

Figure S3. *^{1}H-NMR spectra of 3-methyl-2,4-pentanedione in d4-methanol.*

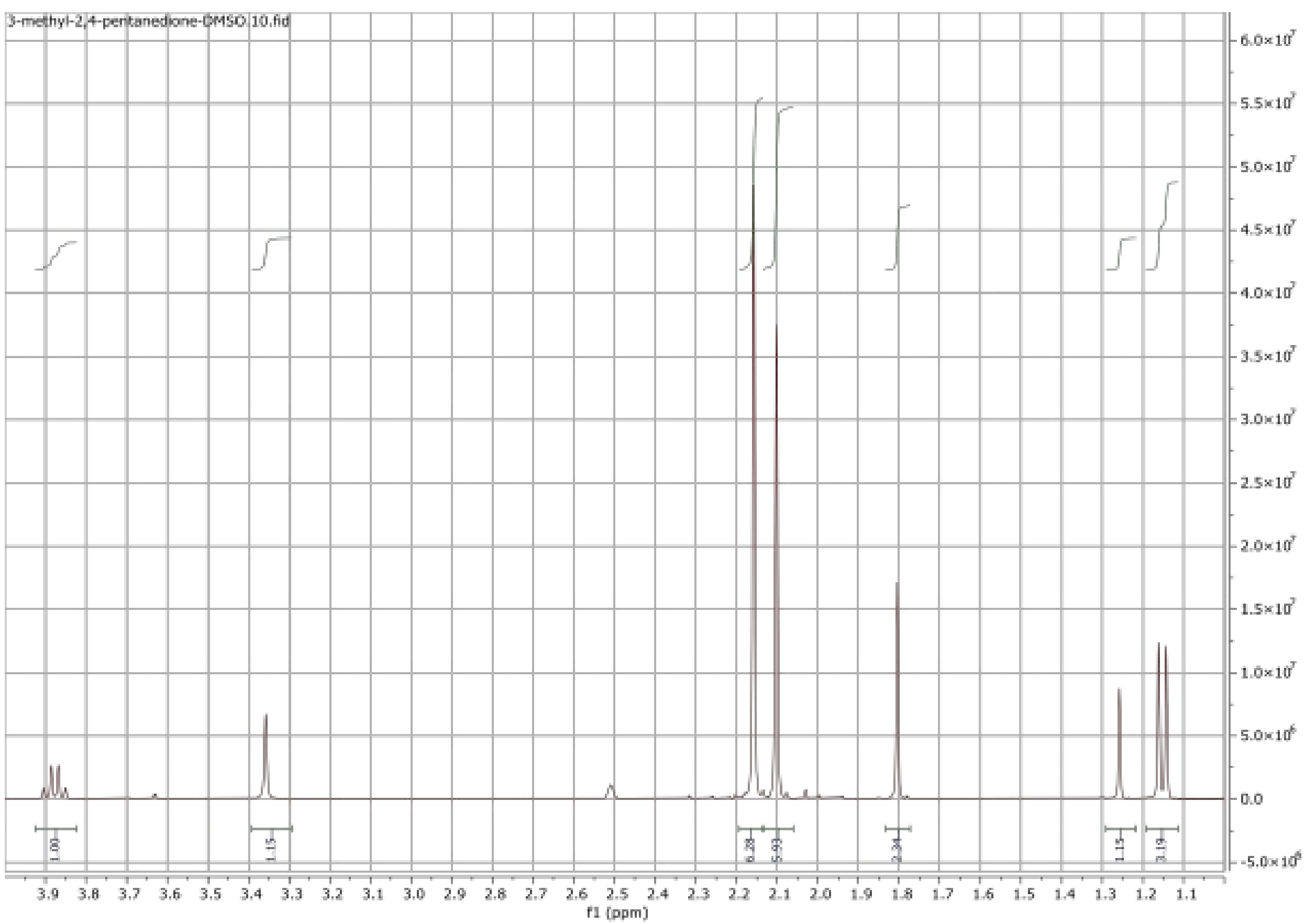

Figure S4. *^{1}H-NMR spectra of 3-methyl-2,4-pentanedione in d1-chloroform.*

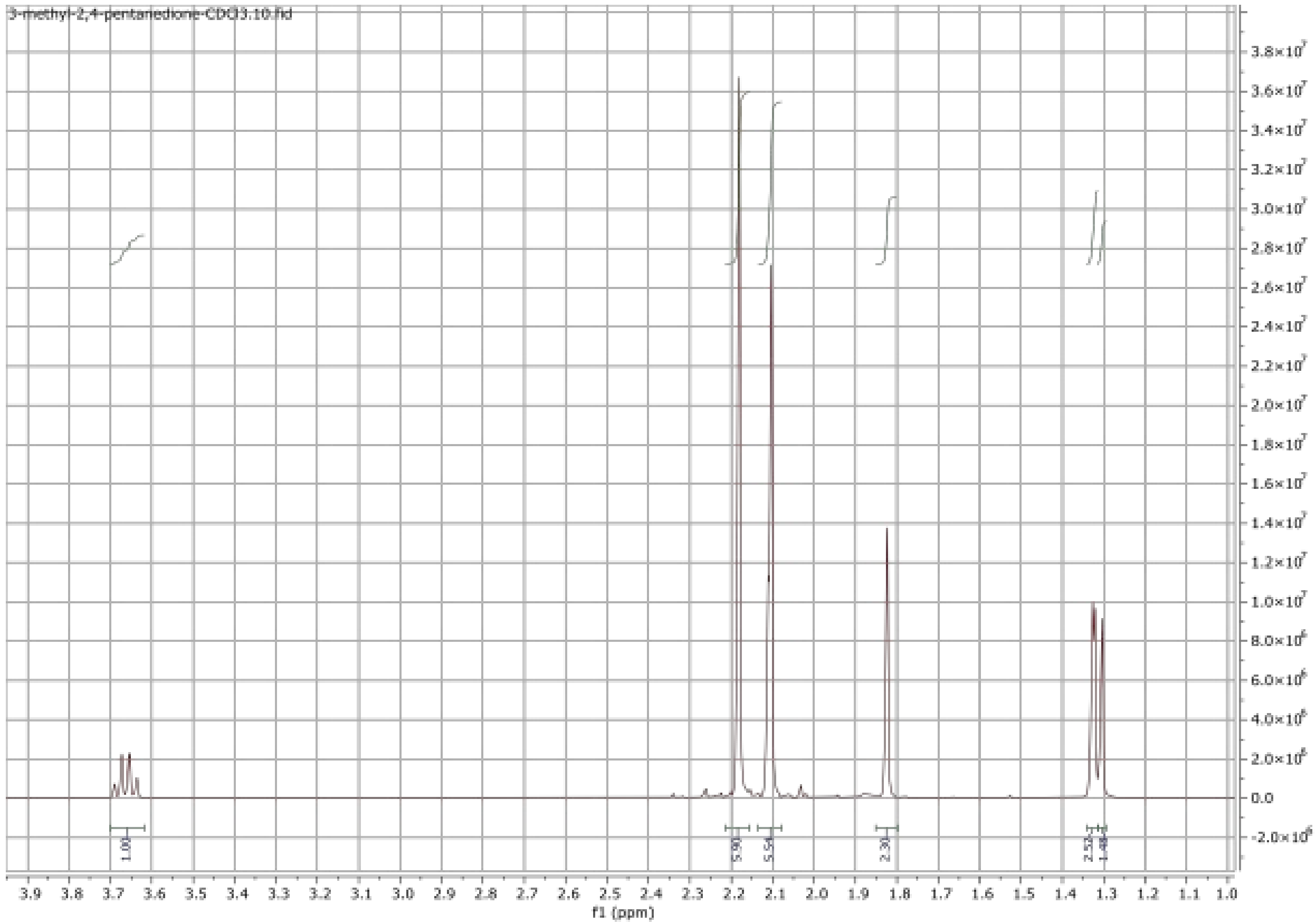

Figure S5. *^{1}H-NMR spectra of 3-methyl-2,4-pentanedione in d6-dimethylsulfoxide.*

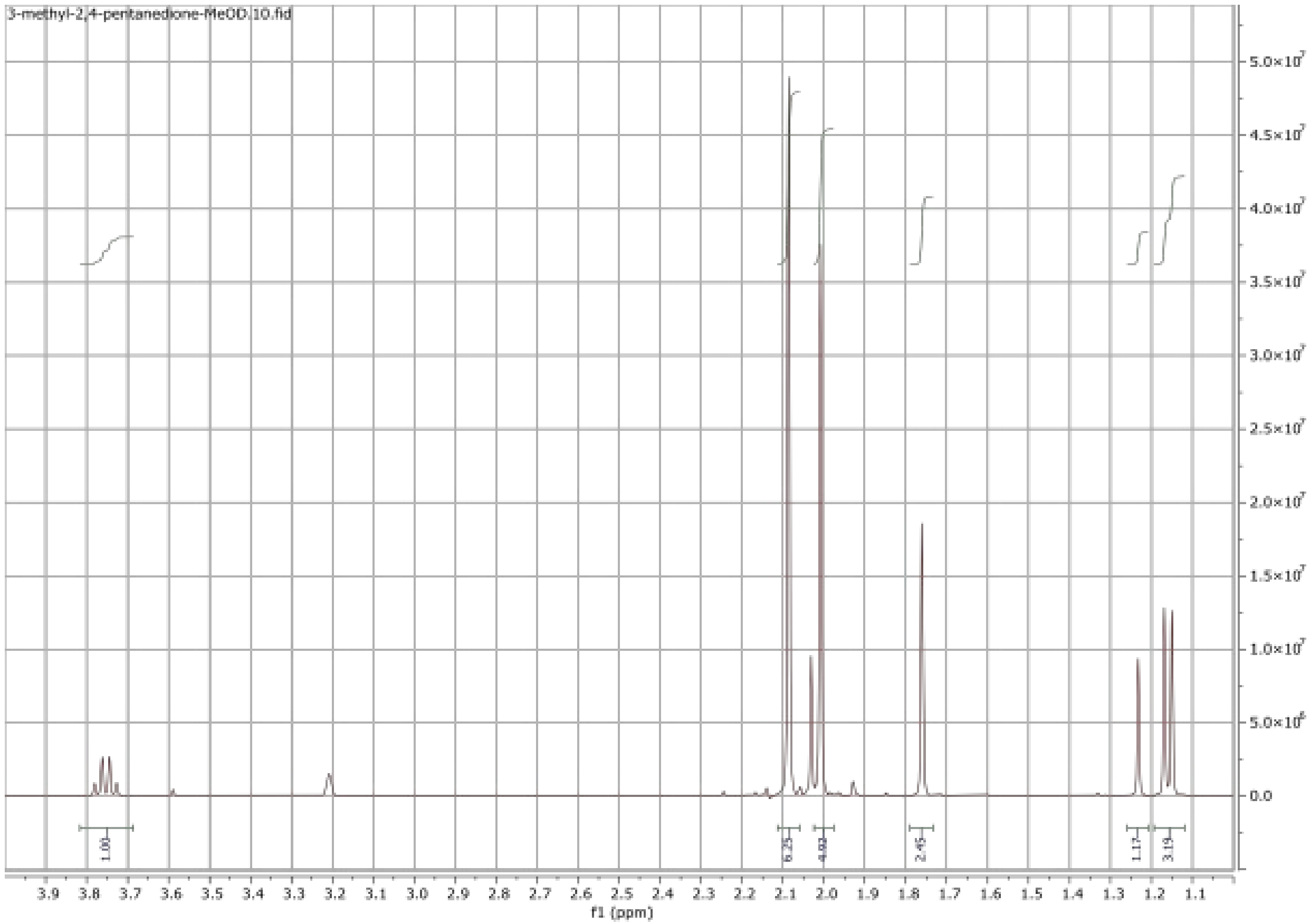

Figure S6. *^{1}H-NMR spectra of 3-chloroacetylacetone in d4-methanol.*

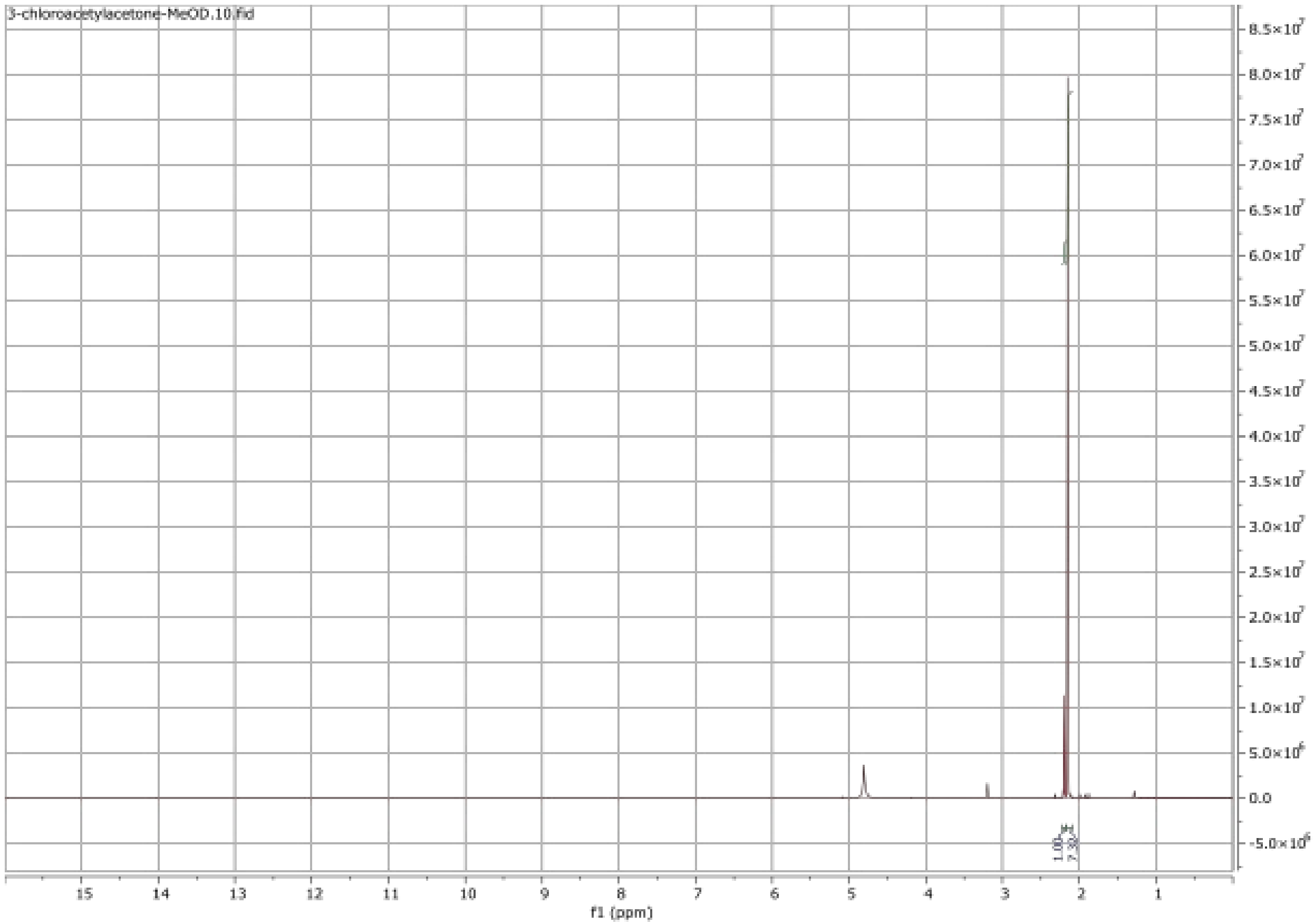

Figure S7. *^{1}H-NMR spectra of 3-chloroacetylacetone in d1-chloroform.*

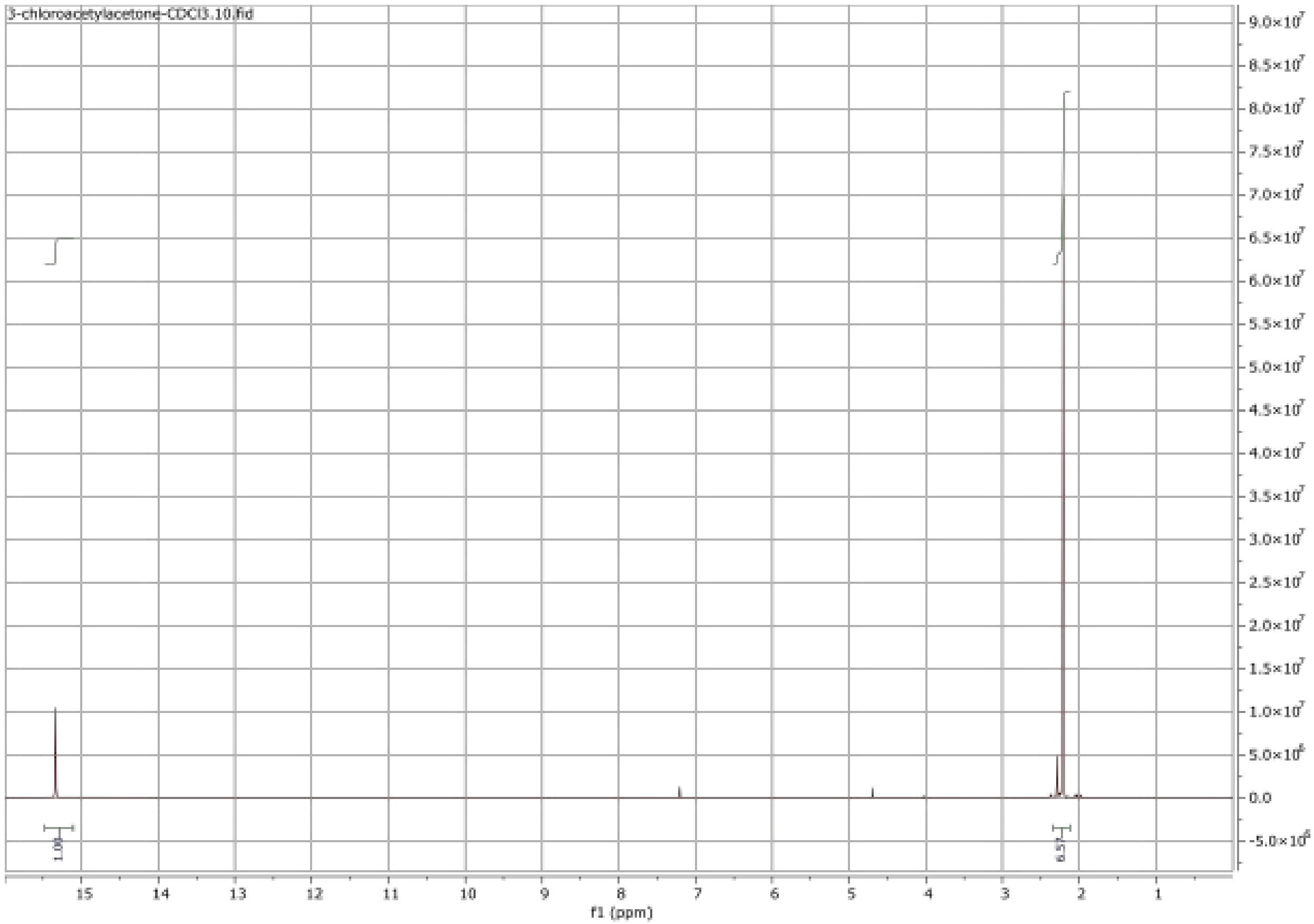

Figure S8. *^{1}H-NMR spectra of 3-chloroacetylacetone in d6-dimethylsulfoxide.*

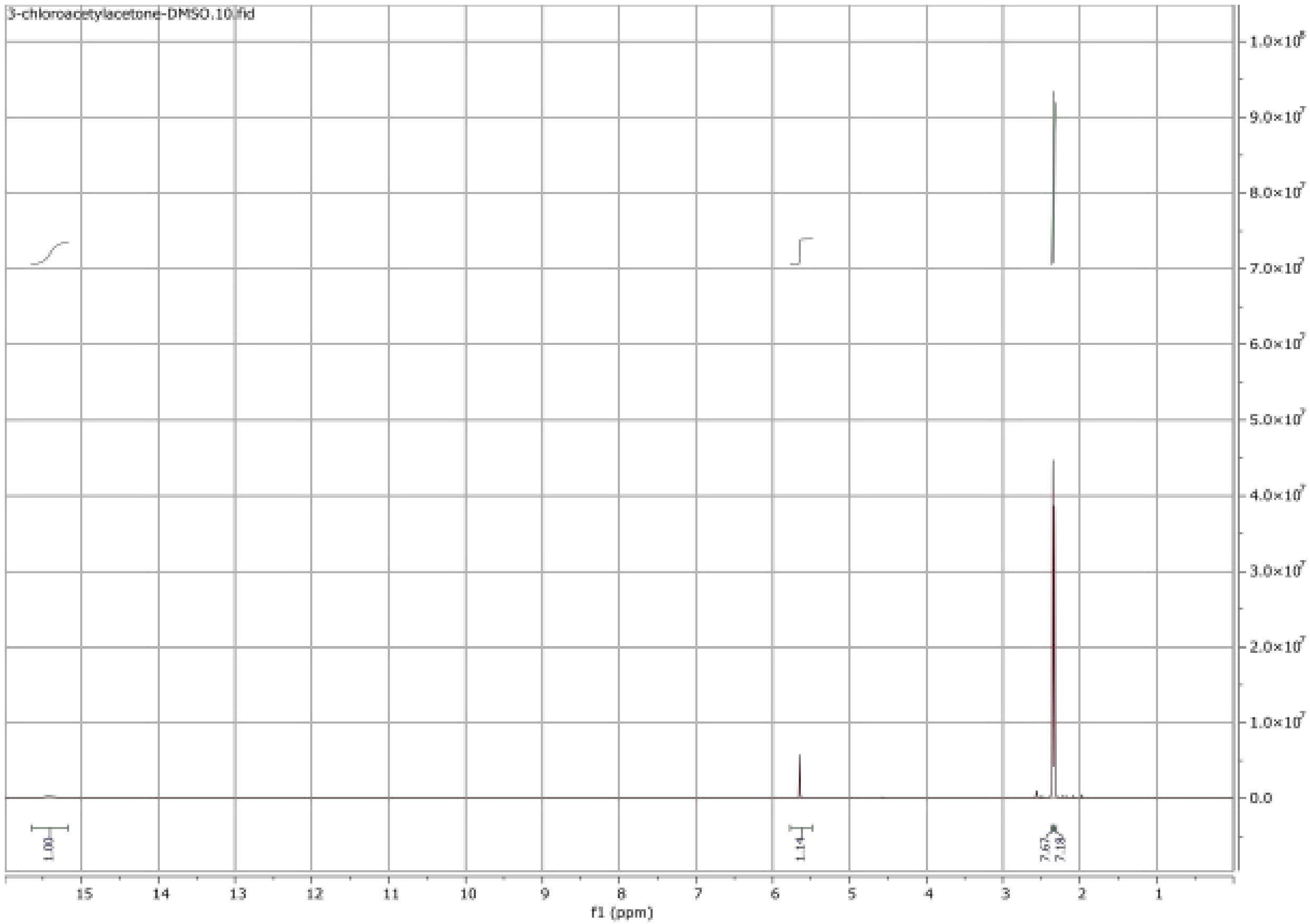

Figure S9. *^{1}H-NMR spectra of 3-phenyl-2,4-pentanedione in d4-methanol.*

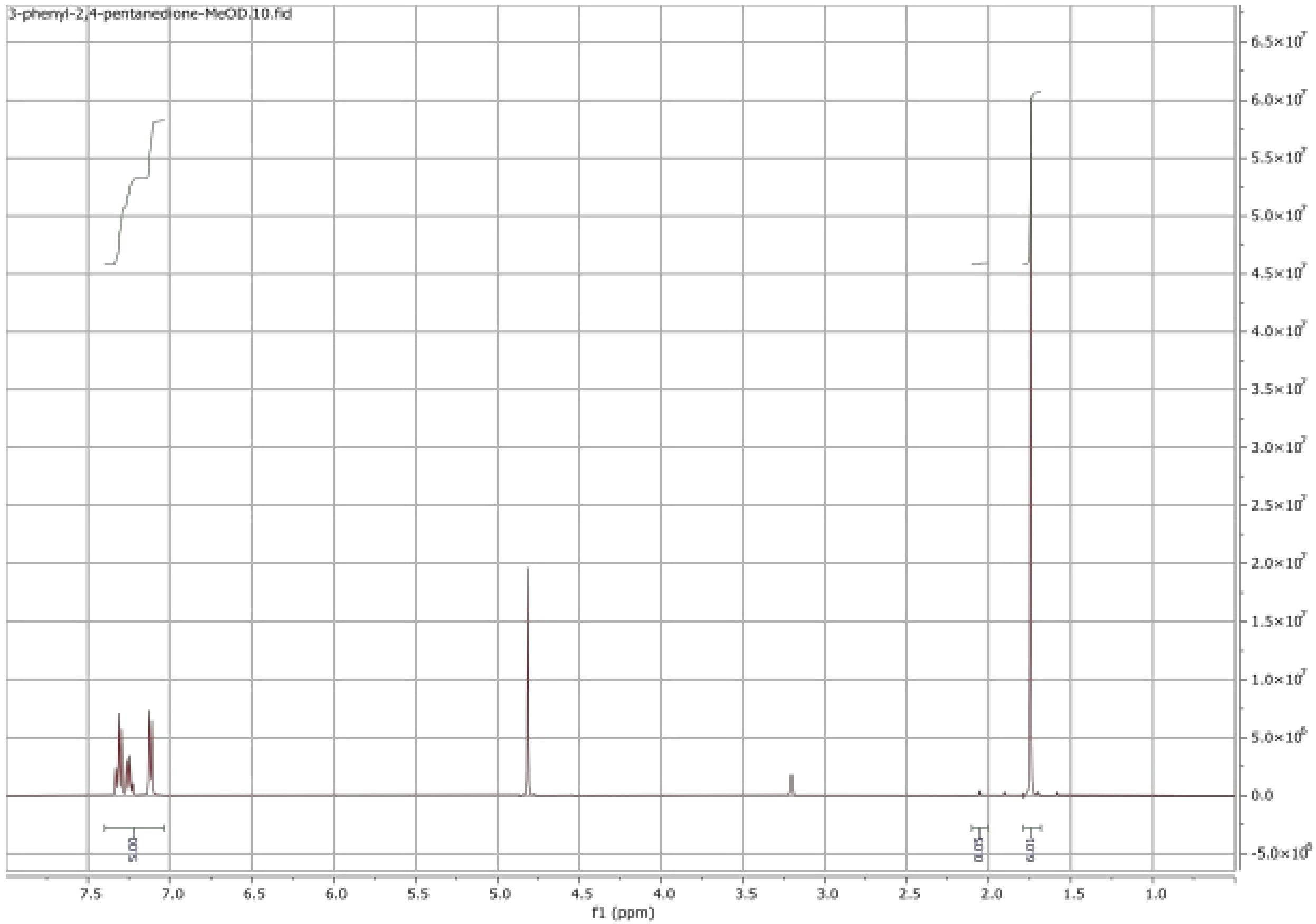

Figure S10. ¹H-NMR spectra of 3-phenyl-2,4-pentanedione in d1-chloroform.

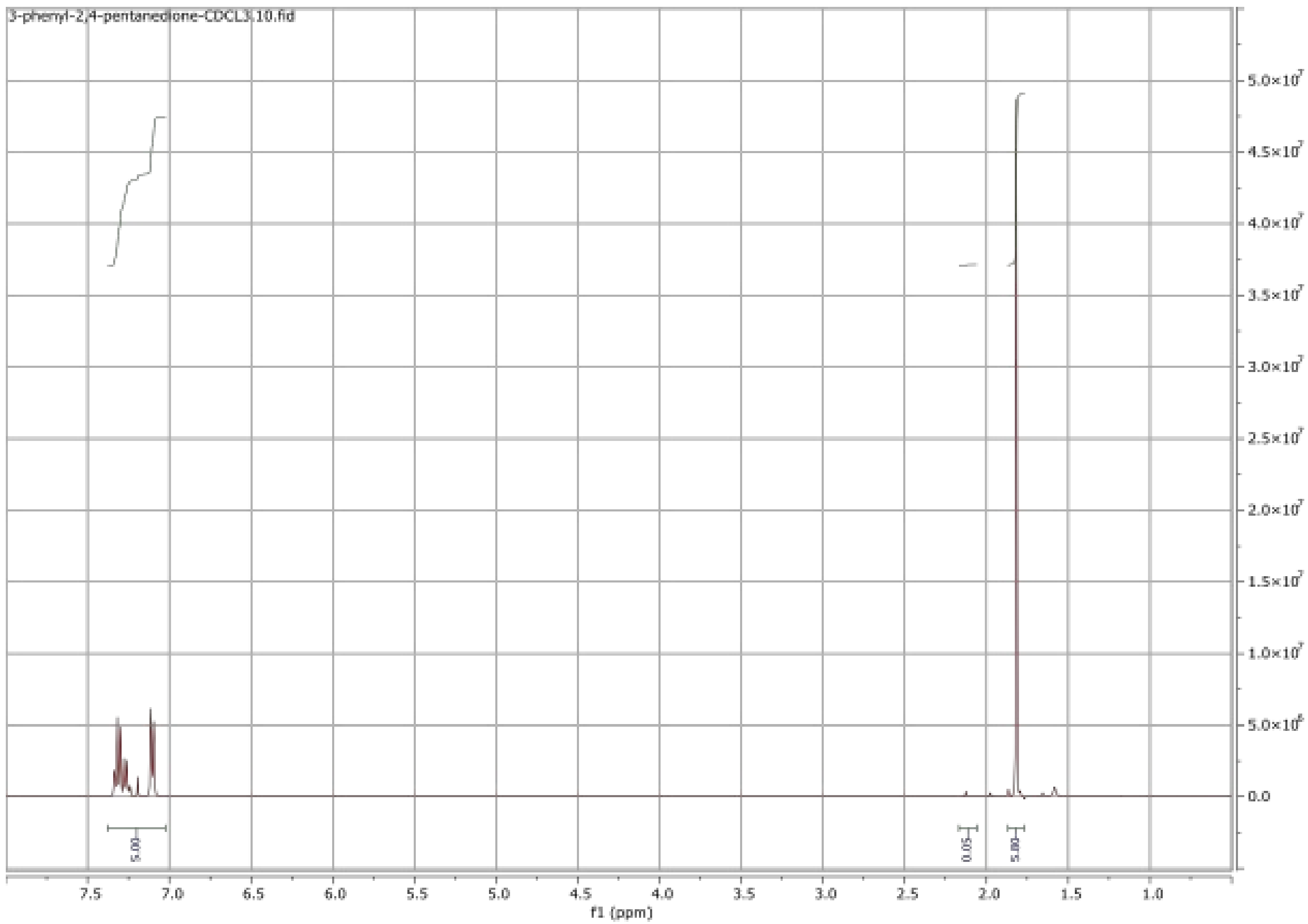

Figure S11. *^{1}H-NMR spectra of 3-phenyl-2,4-pentanedione in d6-dimethylsulfoxide.*

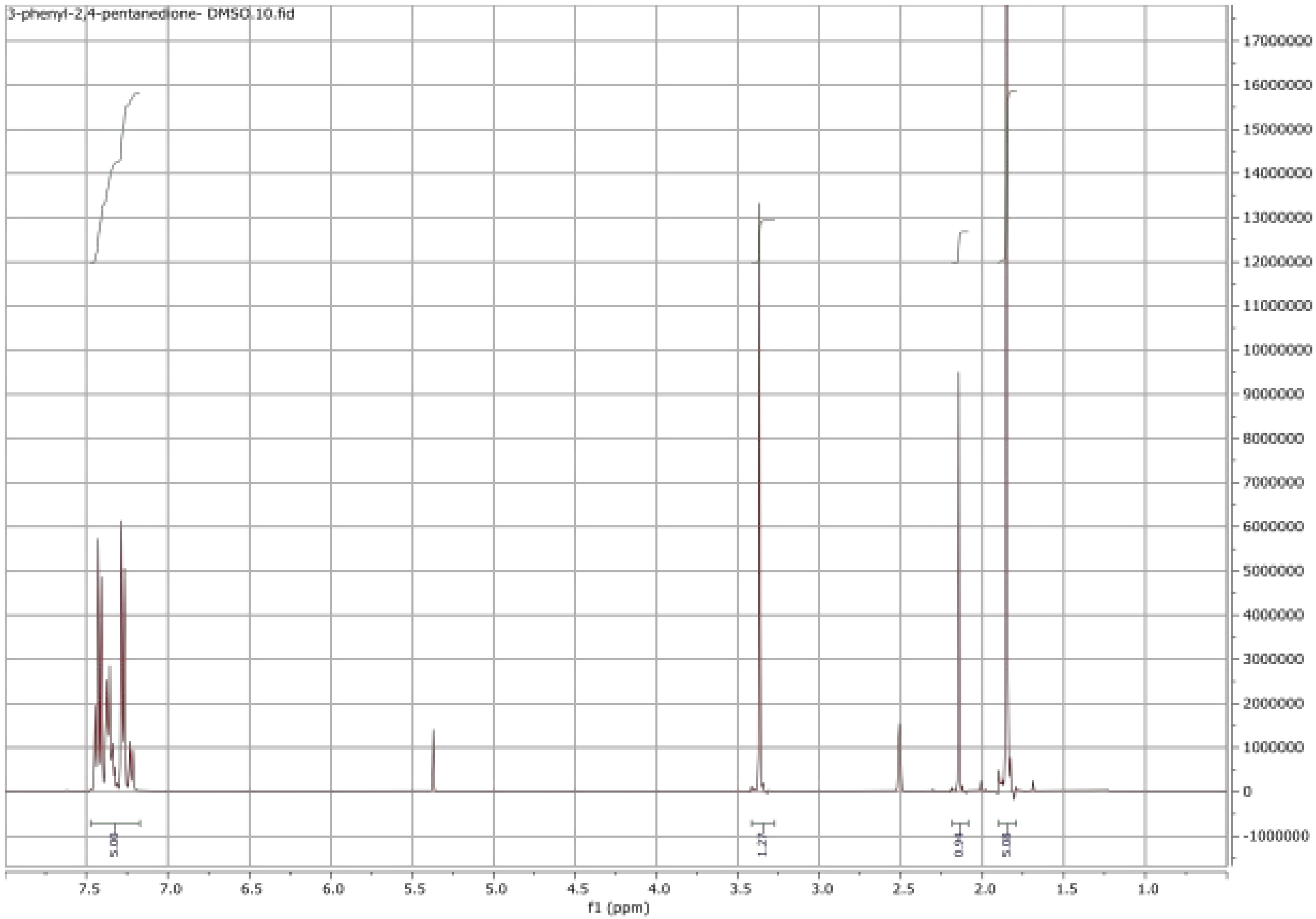

Figure S12. Estimation of Experimental Uncertainty for ^{1}H-NMR Analysis.

Experimental LOQ was estimated by analysis of 10, 20 and 100 ppm samples of 3-phenyl-2,4-pentanedione in $CDCl_3$. The signal to noise ratio was determined by use of the SNR tool in Mnova. Based on this analysis the LOQ (signal 3-fold enhanced over baseline) for 3-phenyl-2,4-pentanedione is 2.3 ppm (0.00023%).

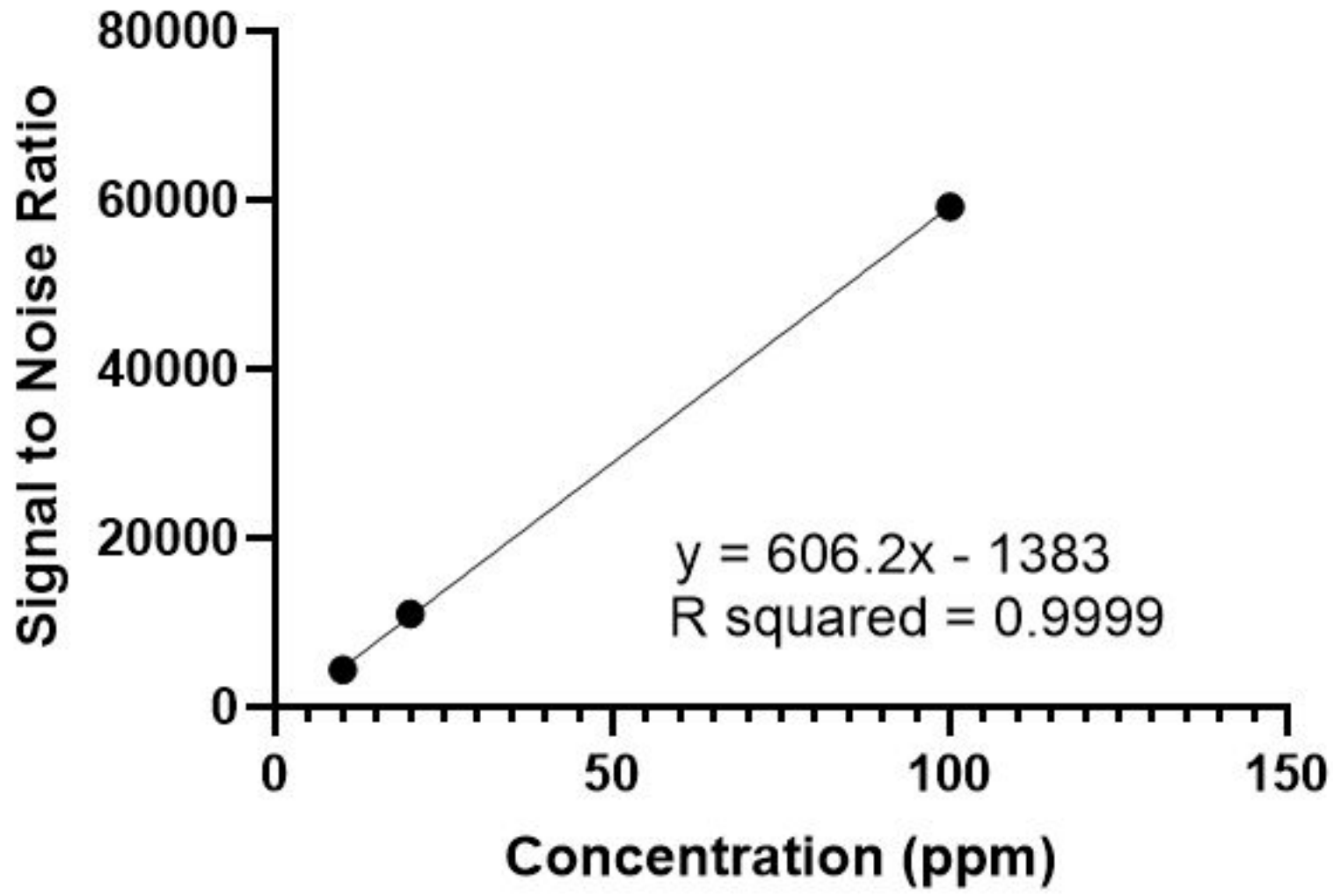

Figure S13. *Compound E1in explicit methanol solvent*

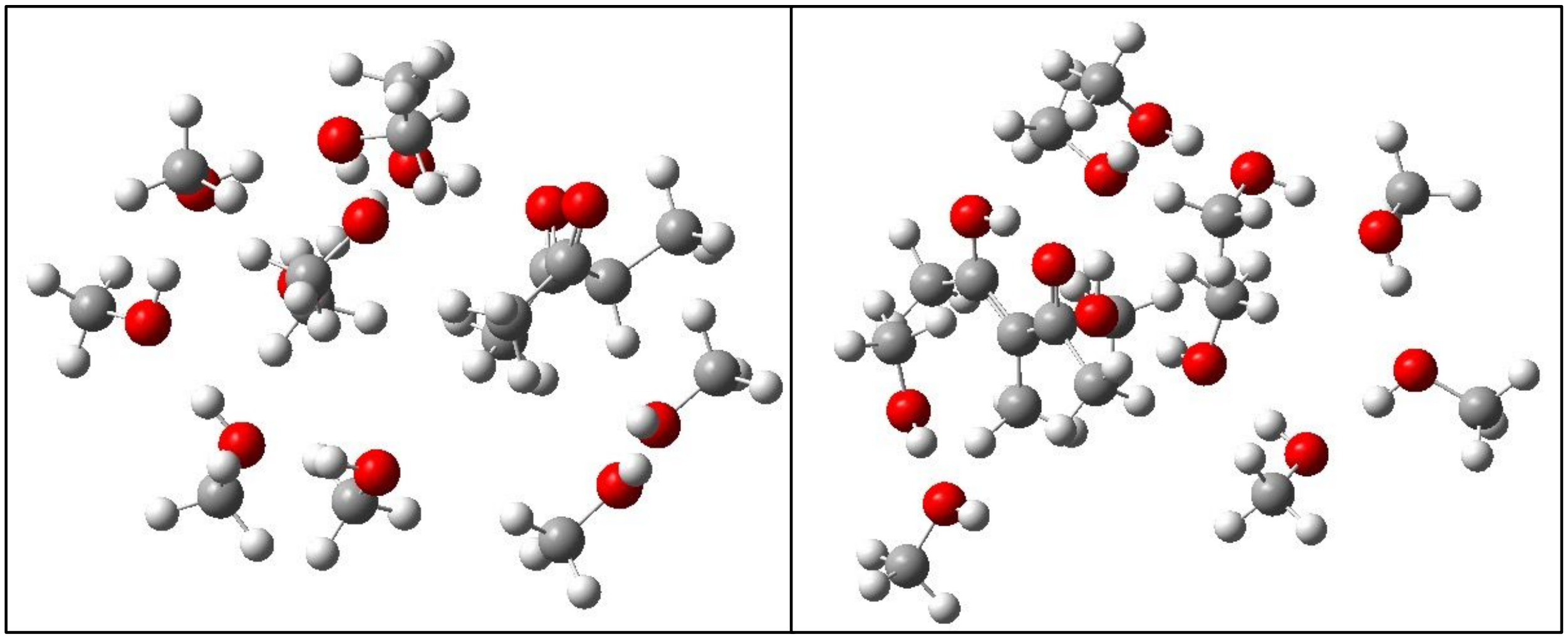

Figure S14. *Compound E1 in explicit DMSO solvent*

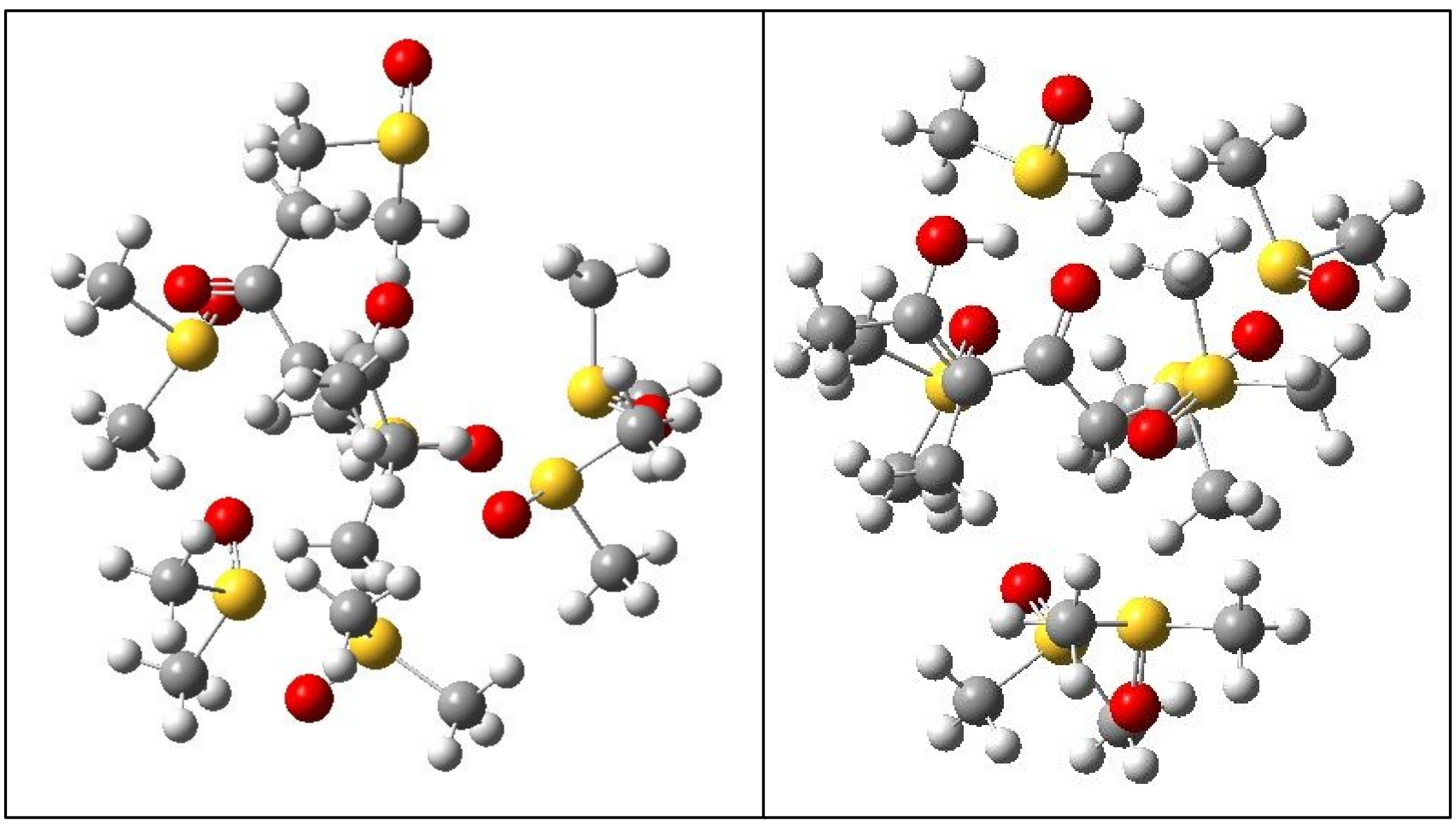

Figure S15. *Compound E1 in explicit chloroform solvent*

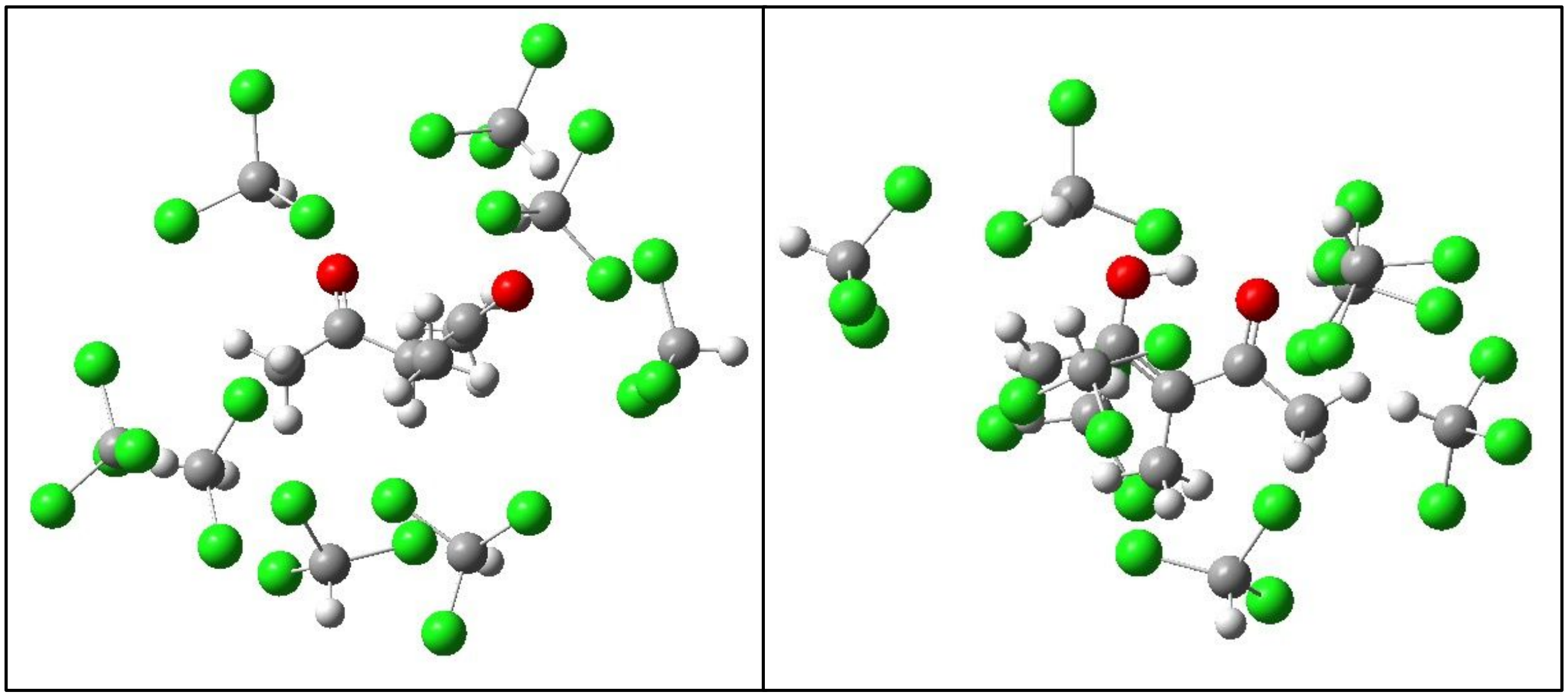

Figure S16. *Compound E2 in explicit methanol solvent*

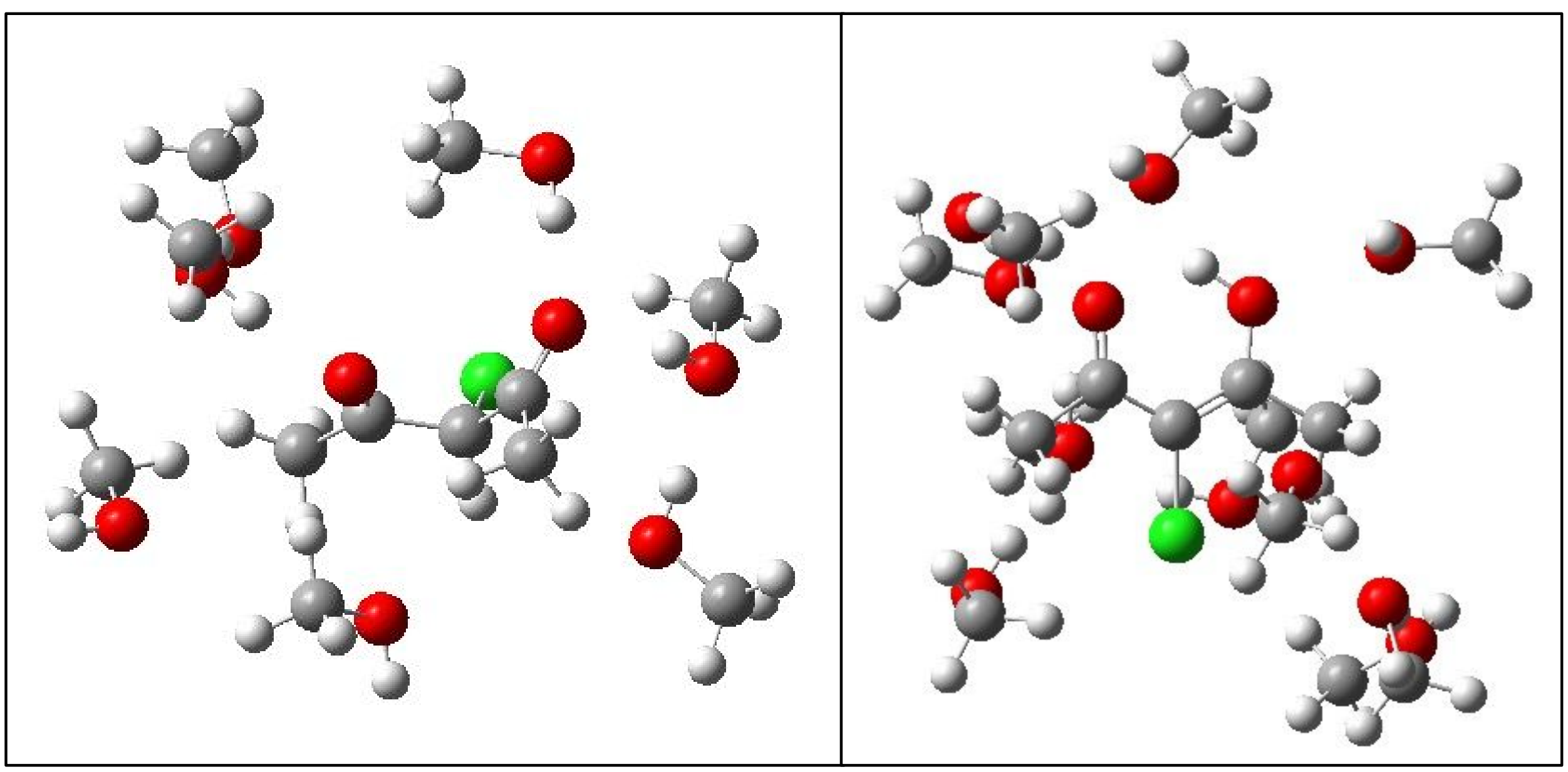

Figure S17. *Compound E2 in explicit DMSO solvent*

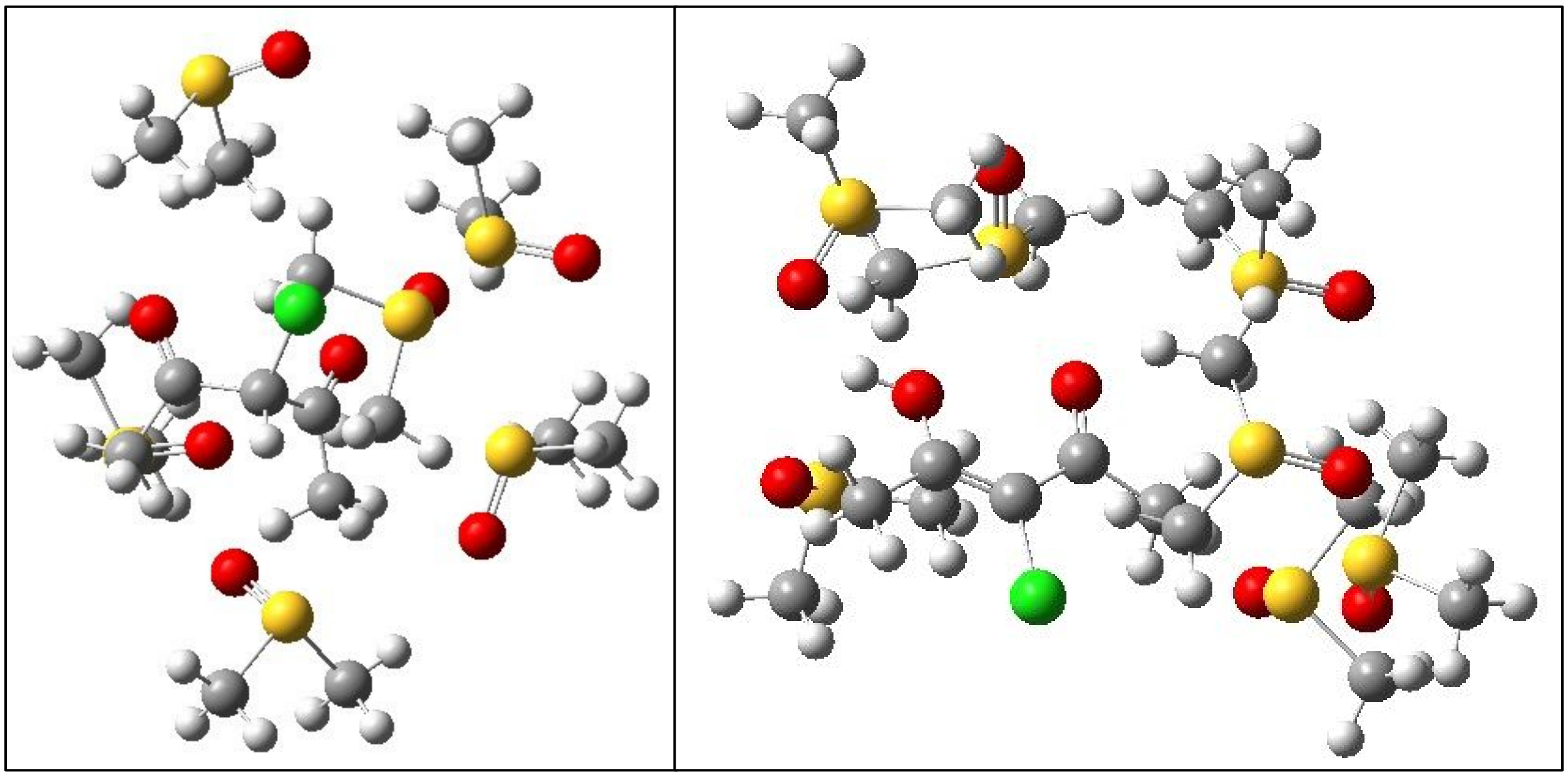

Figure S18. *Compound E2 in explicit chloroform solvent*

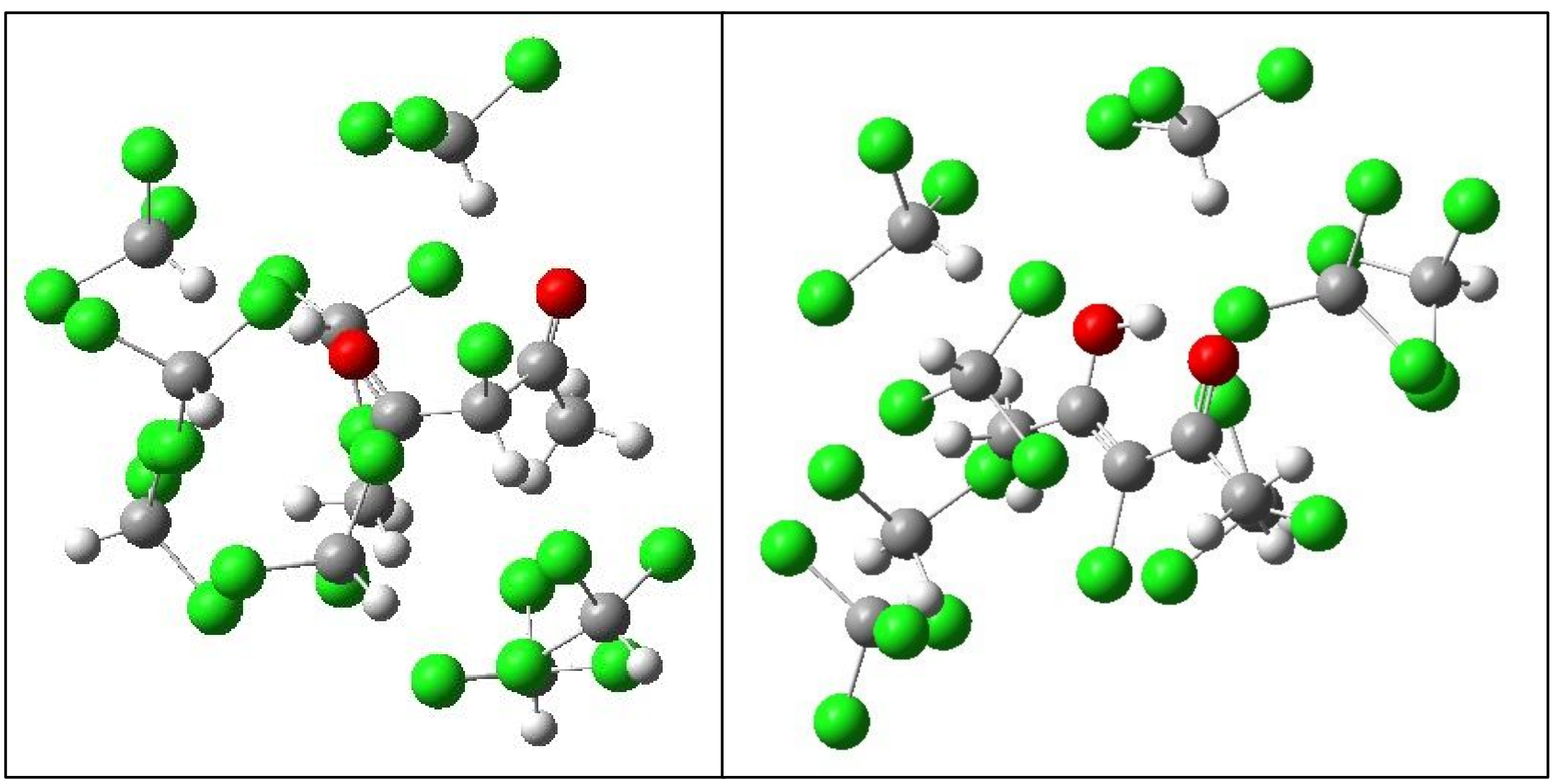

Figure S19. *Compound E3 in explicit methanol solvent*

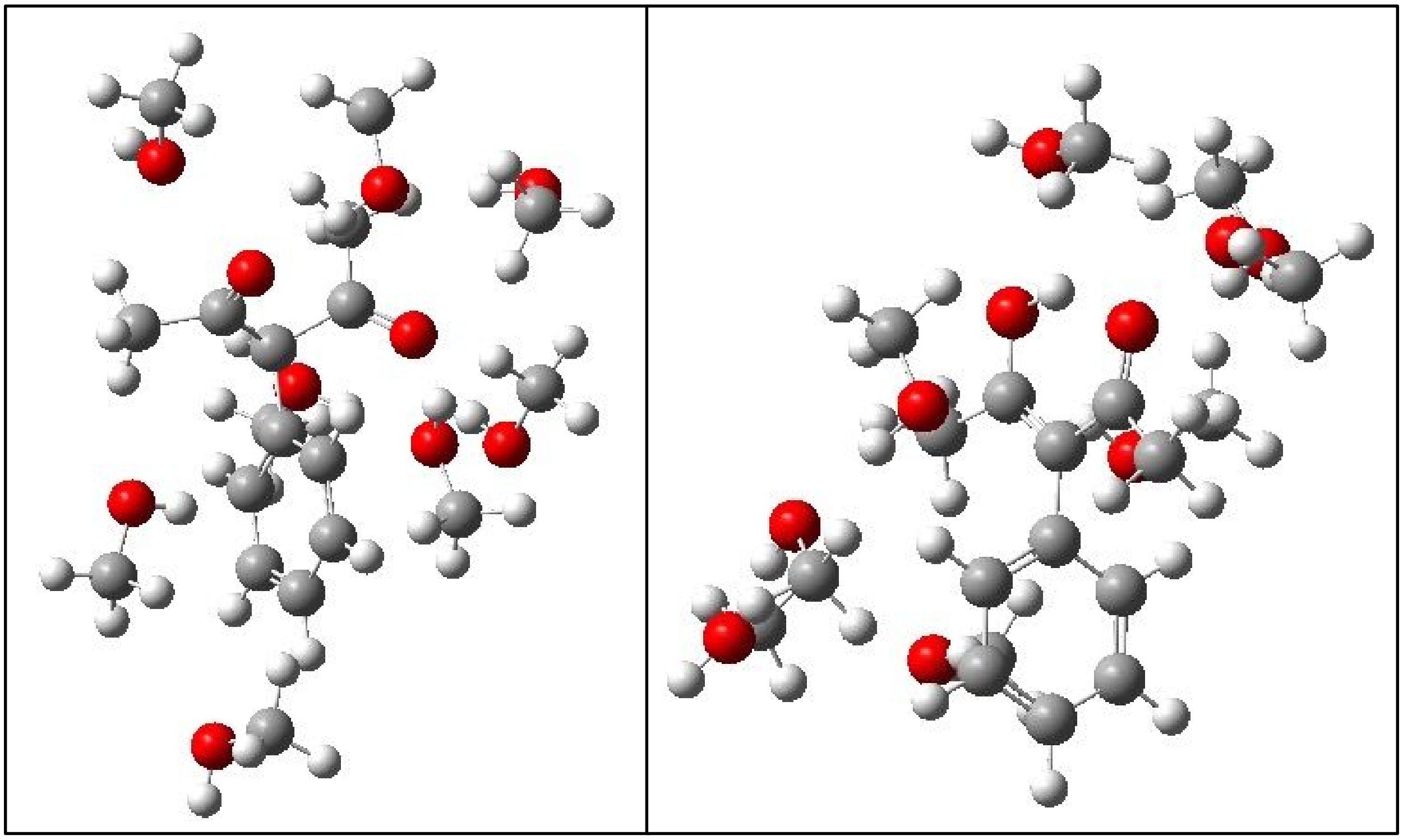

Figure S20. *Compound E3 in explicit DMSO solvent*

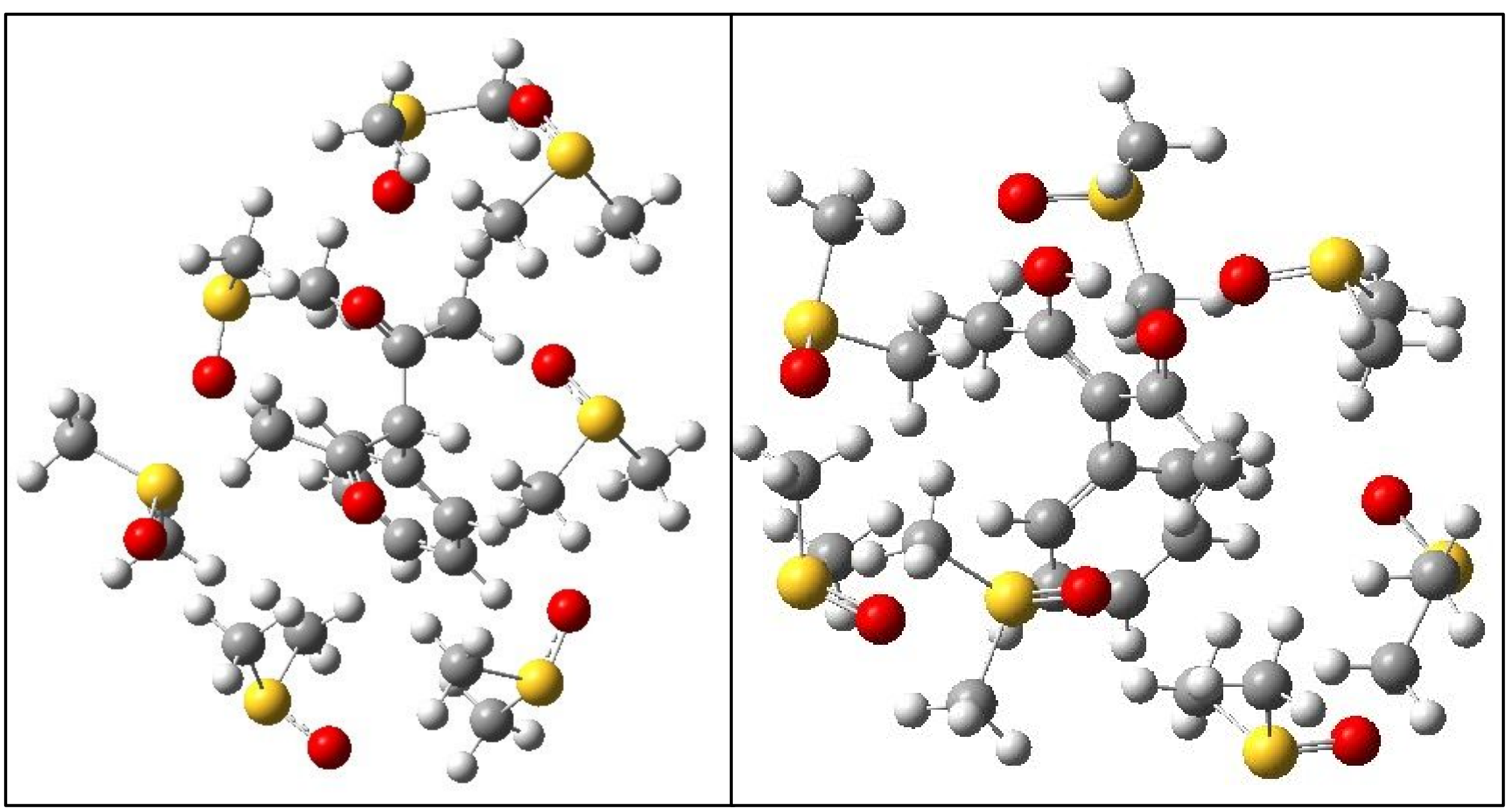

Figure S21. *Compound E3 in explicit chloroform solvent*

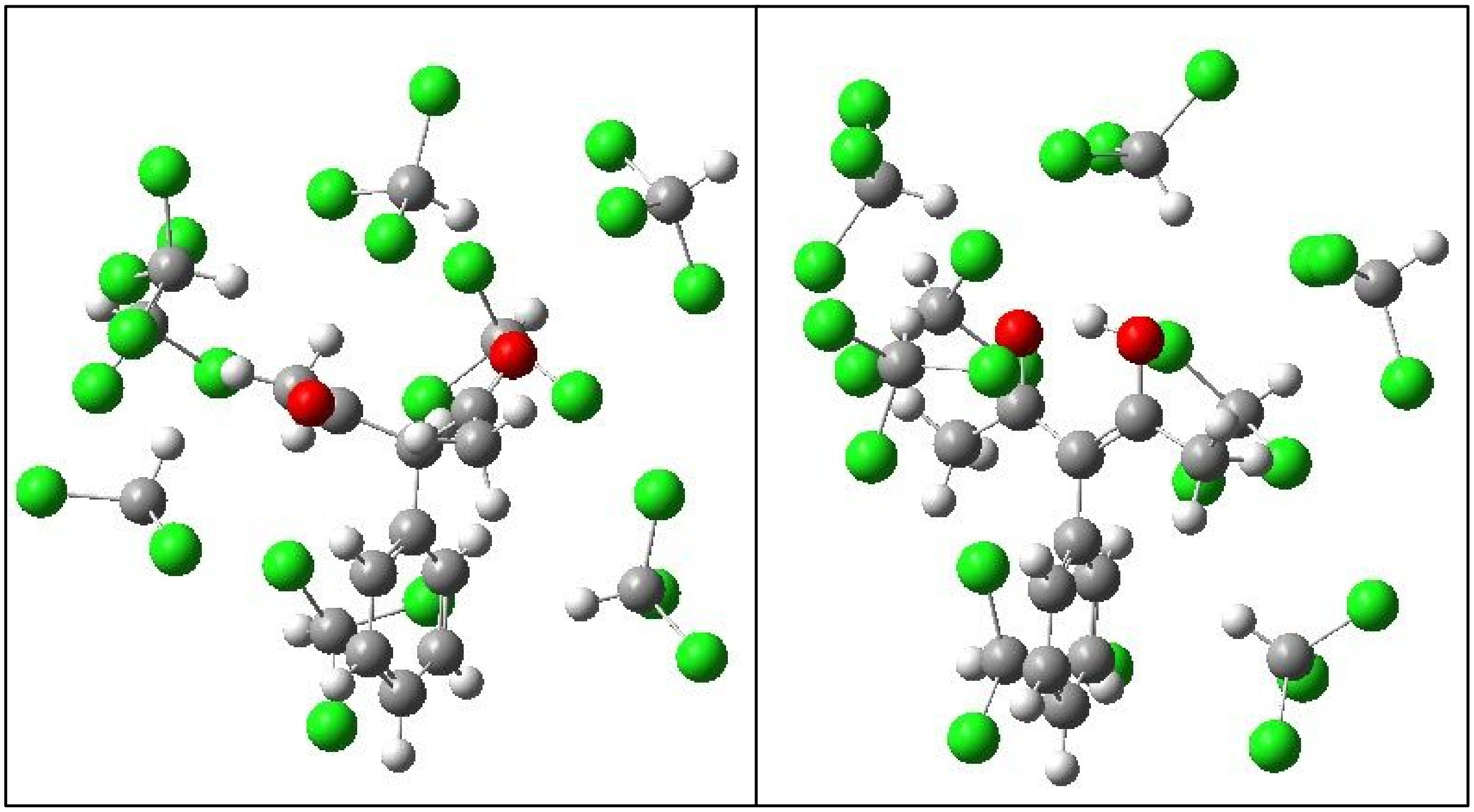

Figure S22. *Compound C1 in explicit methanol solvent*

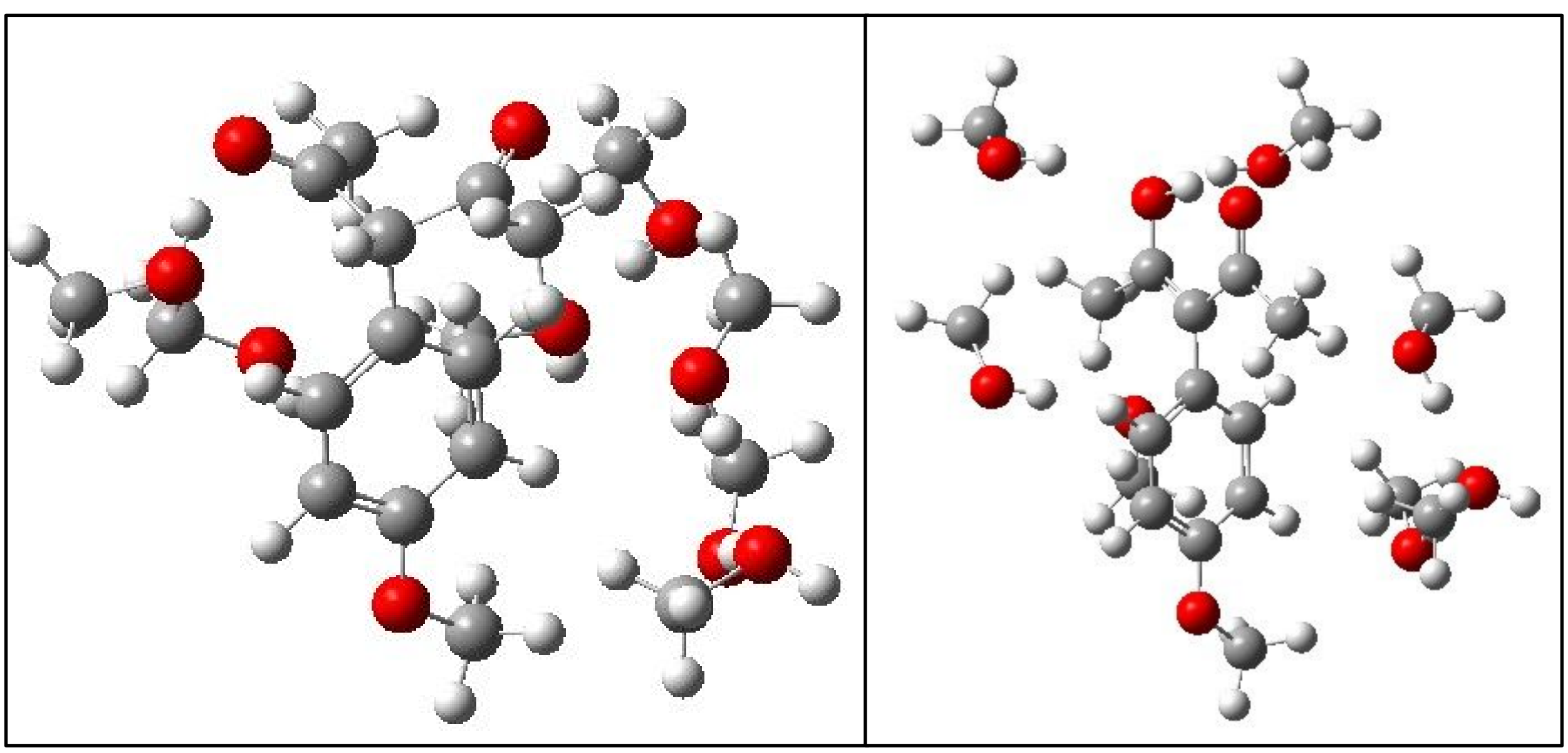

Figure S23. *Compound C1 in explicit DMSO solvent*

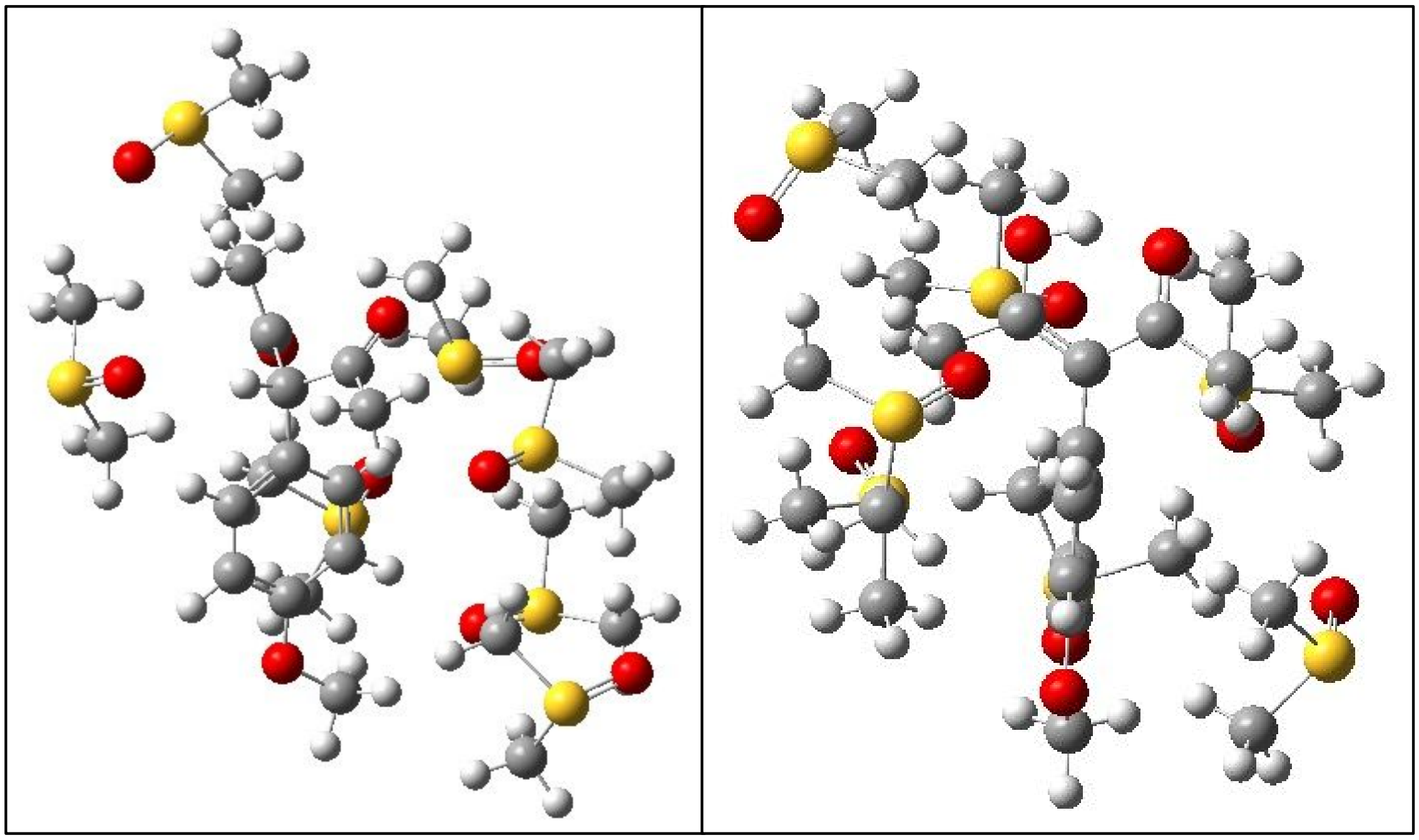

Figure S24. *Compound C1 in explicit chloroform solvent*

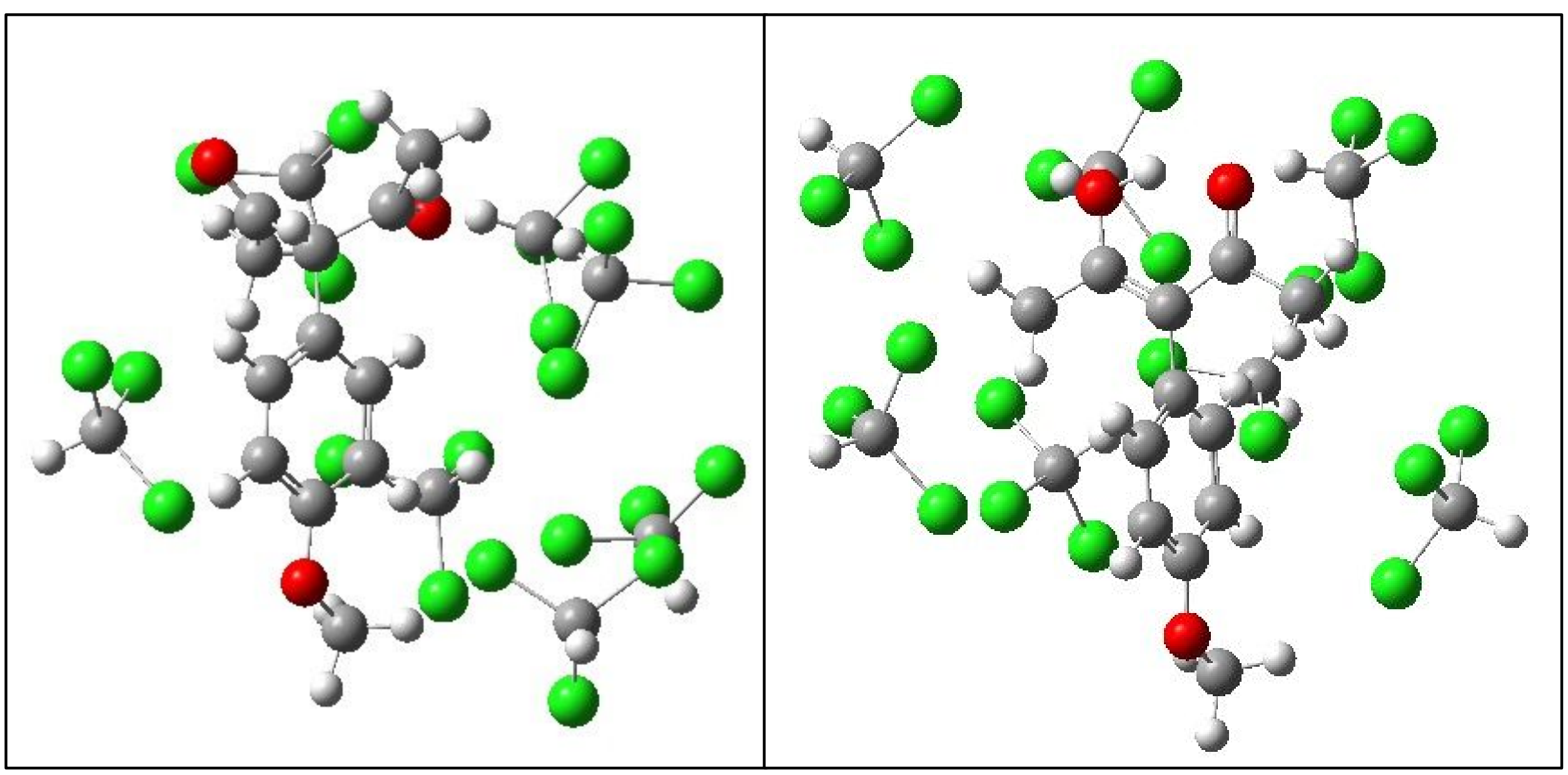

Figure S25. *Compound C2 in explicit methanol solvent*

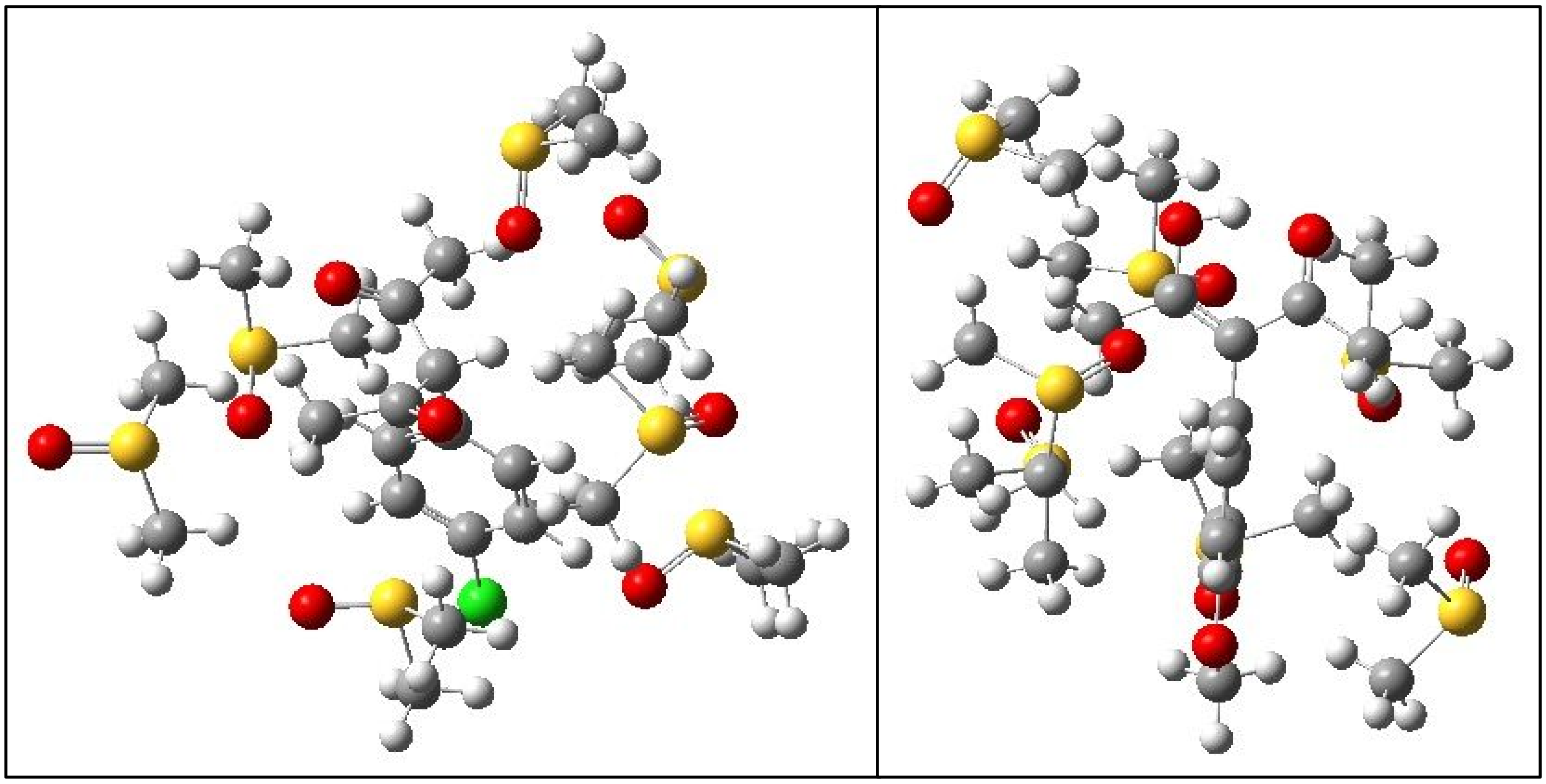

Figure S26. *Compound C2 in explicit DMSO solvent*

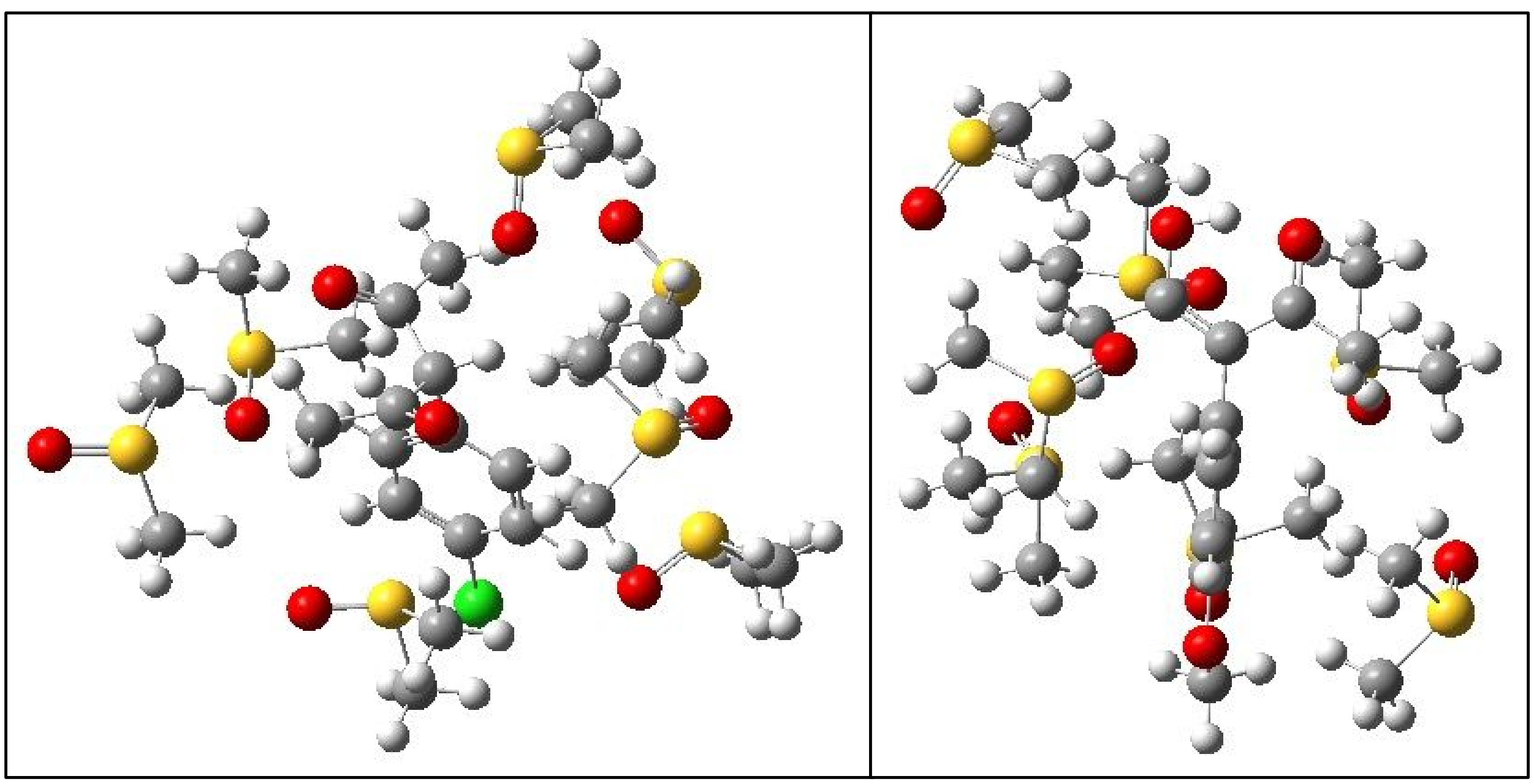

Figure S27. *Compound C2 in explicit chloroform solvent*

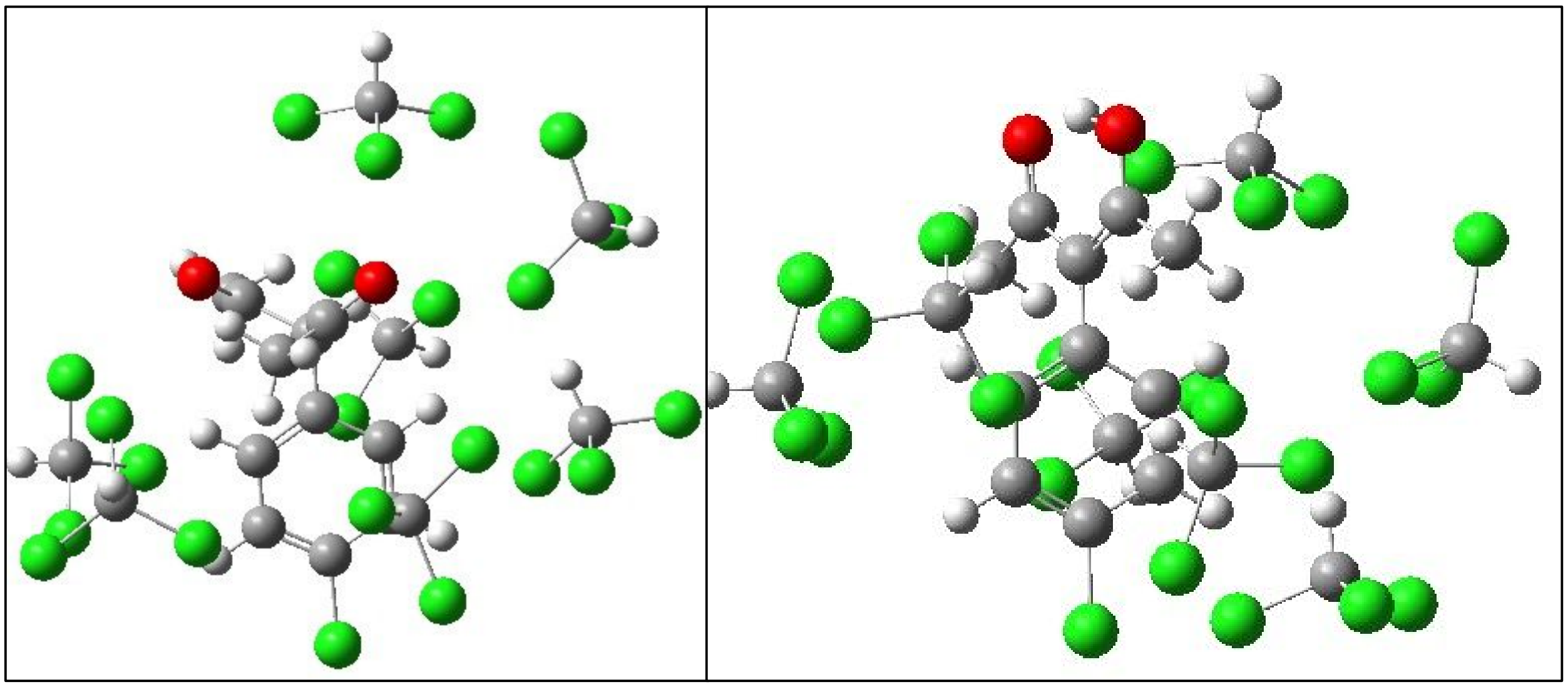

Figure S28. *Compound C3 in explicit methanol solvent*

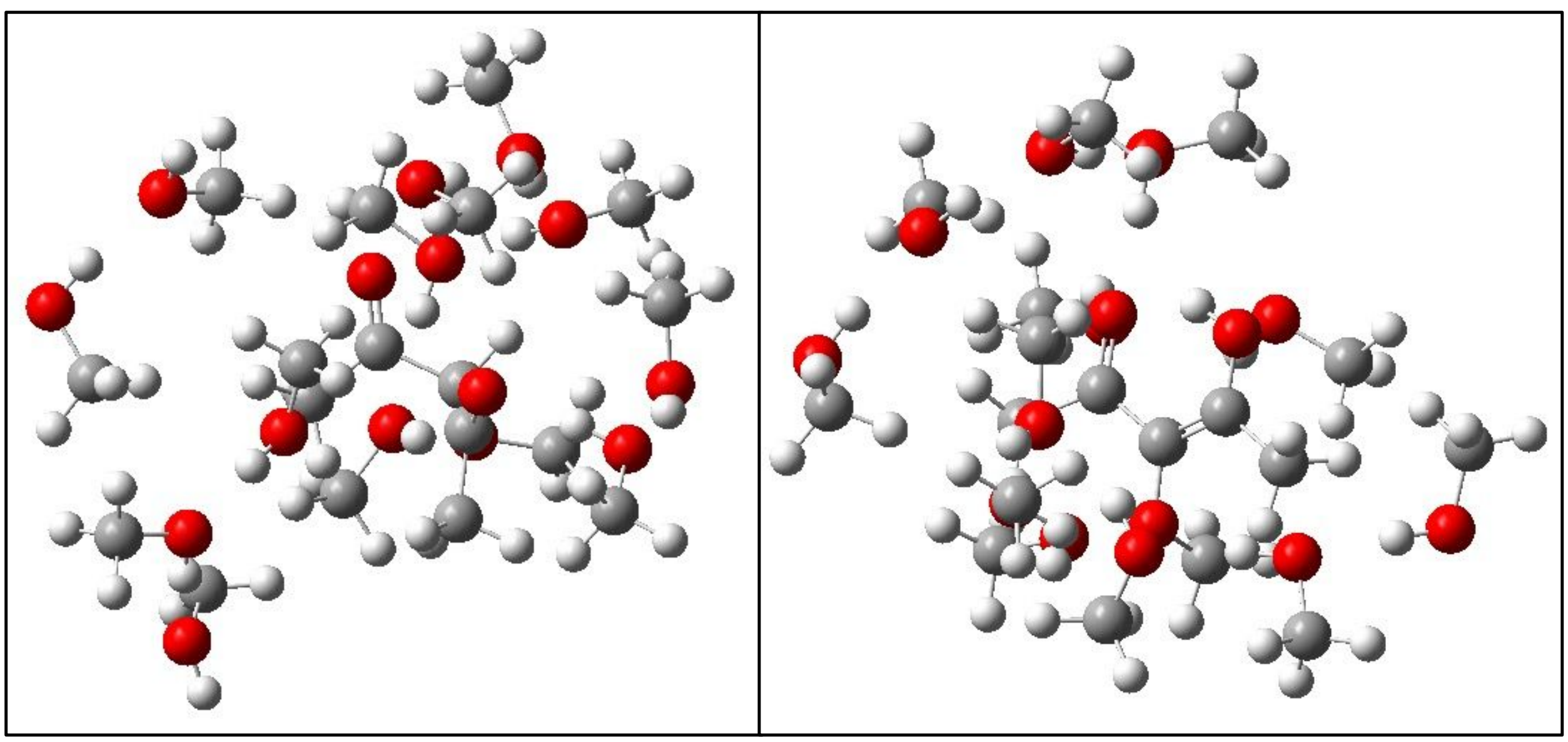

Figure S29. *Compound C3 in explicit DMSO solvent*

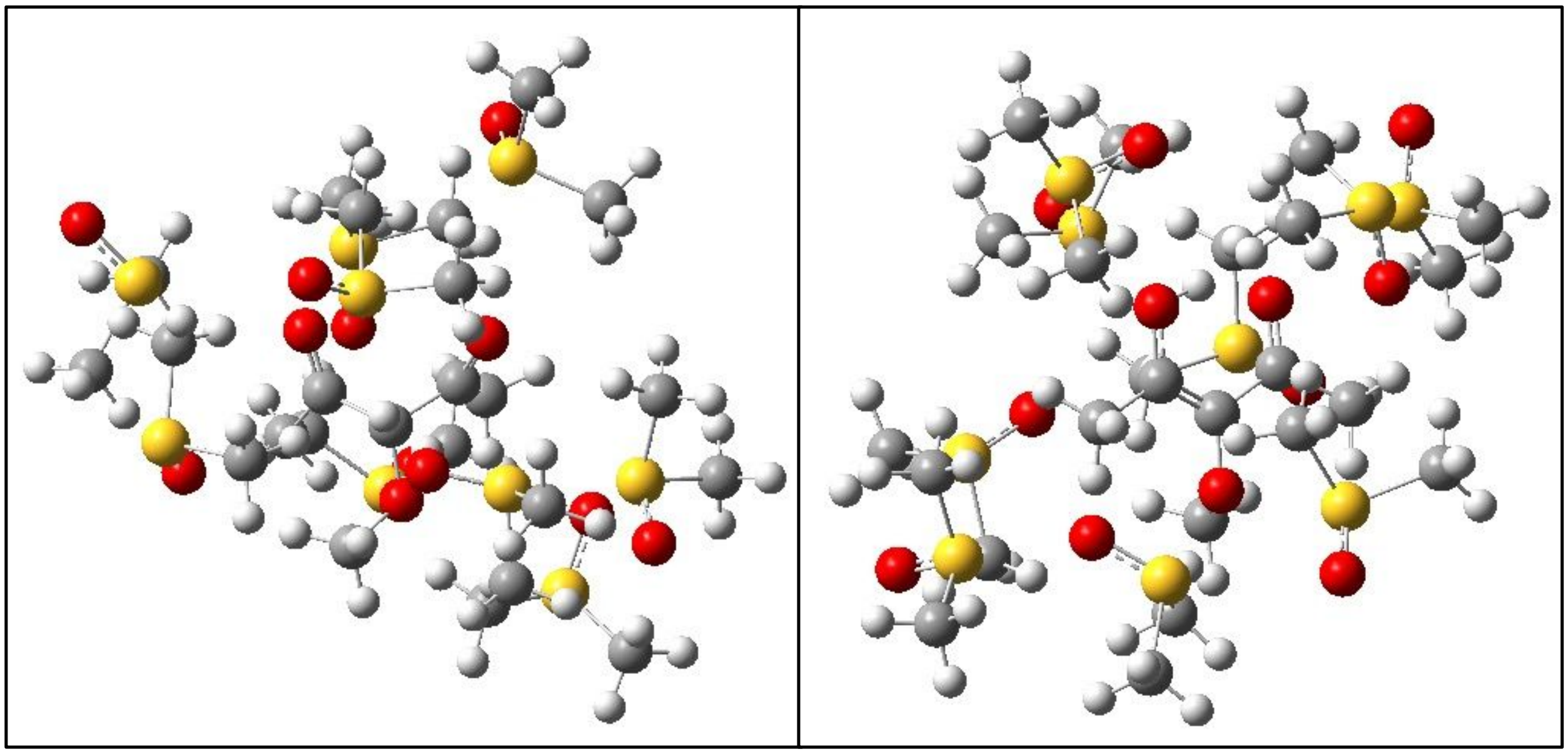

Figure S30. *Compound C3 in explicit chloroform solvent*

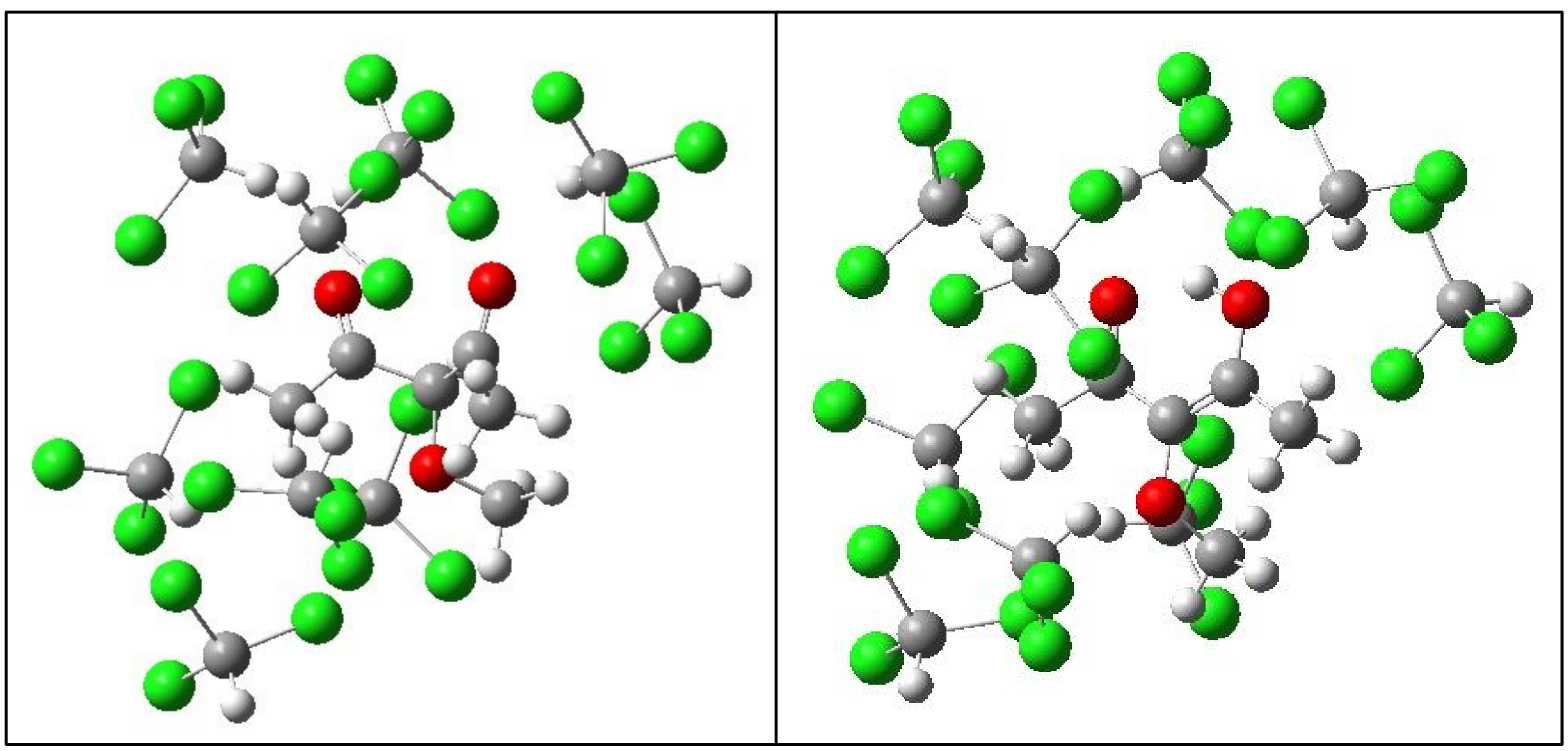

Figure S31. *Compound C4 in explicit methanol solvent*

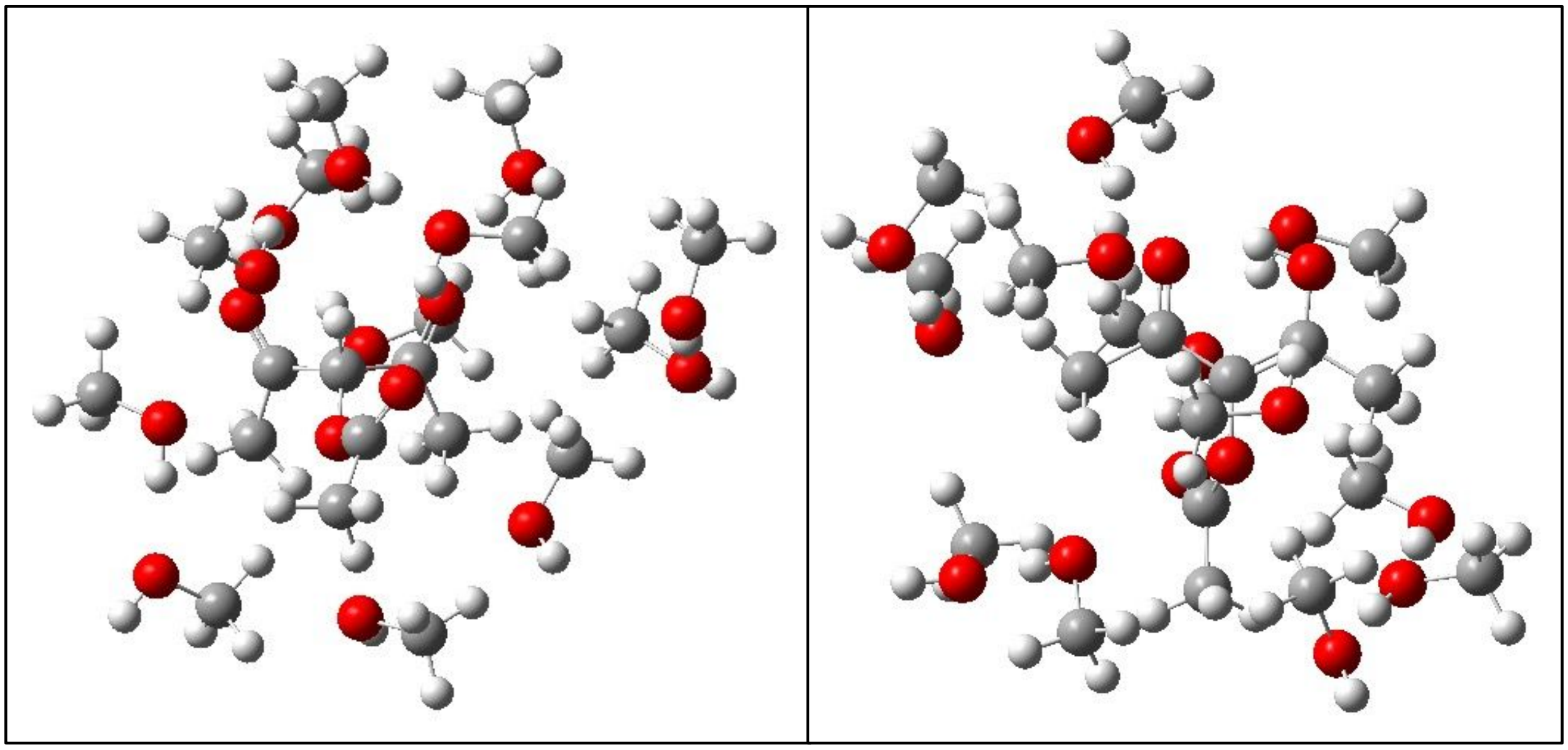

Figure S32. *Compound C4 in explicit DMSO solvent*

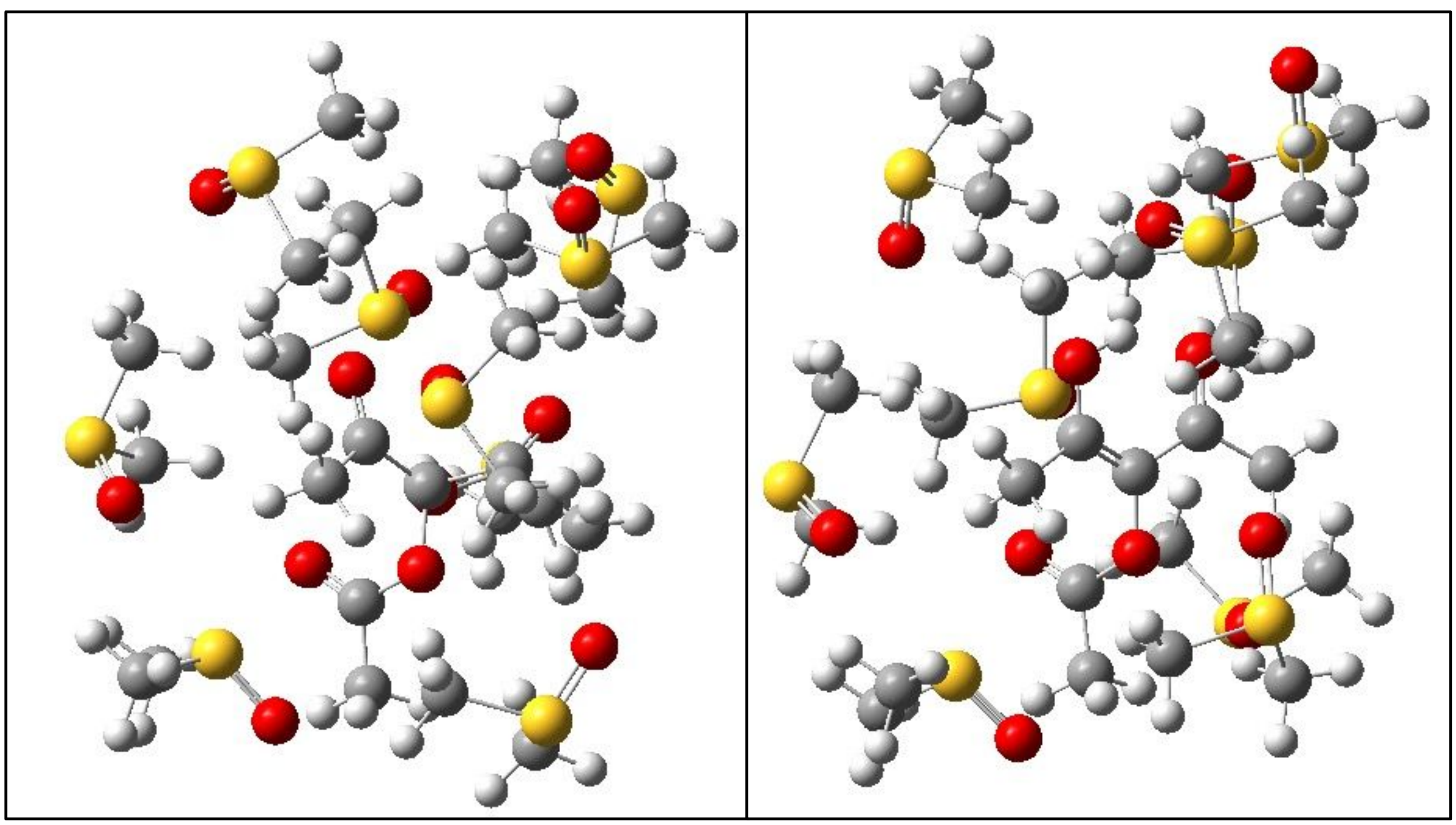

Figure S33. *Compound C4 in explicit chloroform solvent*

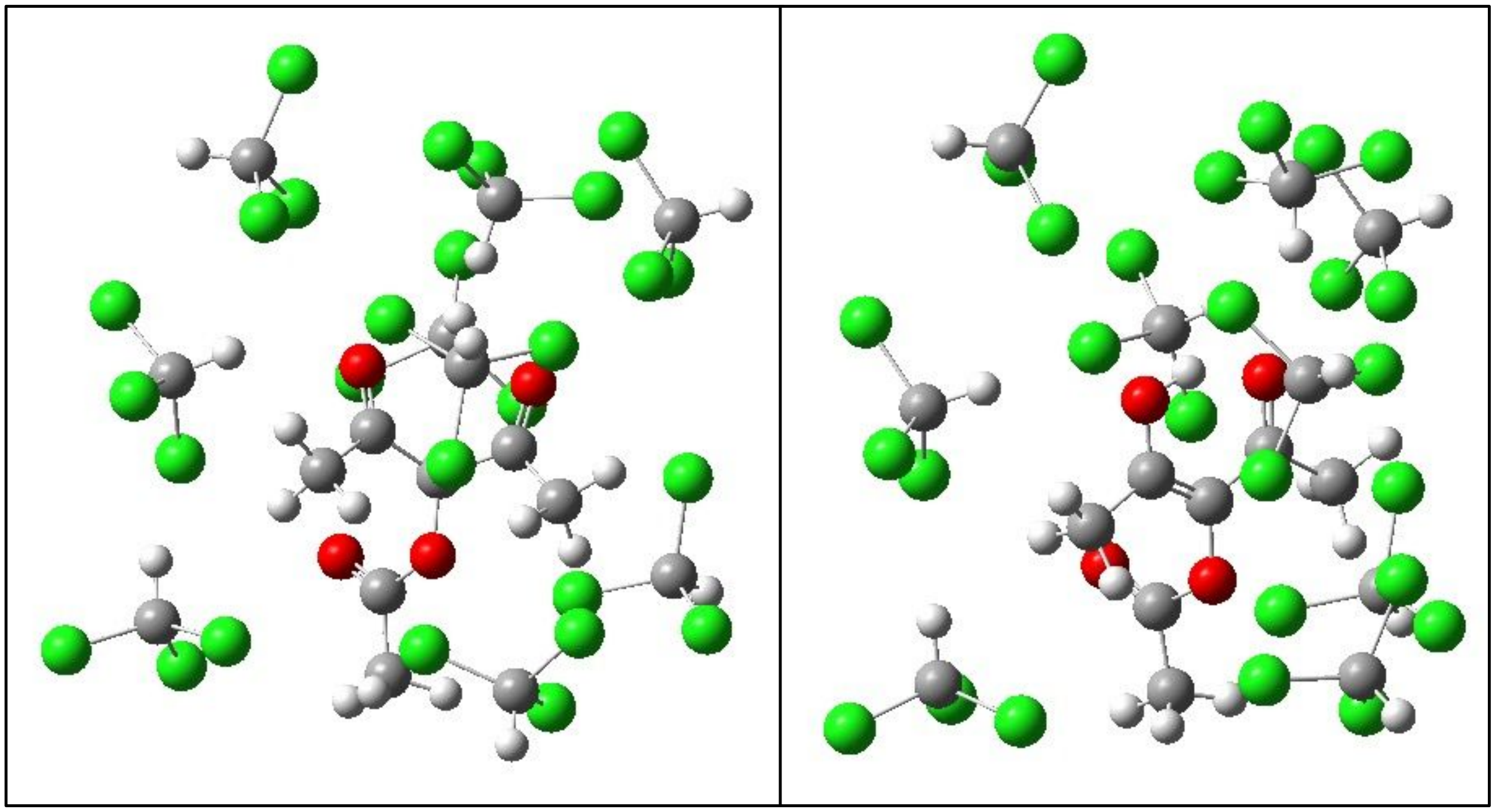

Supplement: Supplementary file 1 [file ao6c03902_si_001.pdf]
